# Supplementary material for: Multi-scale molecular dynamics simulations of enhanced energy transfer in organic molecules under strong coupling
Source: Nat Commun. 2023 Oct 19;14:6613. doi: 10.1038/s41467-023-42067-y (PMC10587084; doi:10.1038/s41467-023-42067-y)
Supplement: Supplementary file 1 — Supplementary Information [file 41467_2023_42067_MOESM1_ESM.pdf]

Supporting Information
for
Enhanced Excitation Energy Transfer under Strong Light-Matter Coupling: Insights from Multi-Scale Molecular Dynamics Simulations

Ilia Sokolovskii,[1,†] Ruth H. Tichauer,[1,2,†] Dmitry Morozov,[1] Johannes Feist,[2] and Gerrit Groenhof[*,1]

[1] *Nanoscience Center and Department of Chemistry, University of Jyväskylä, P.O. Box 35, 40014 Jyväskylä, Finland.*

[2] *Departamento de Física Teórica de la Materia Condensada and Condensed Matter Physics Center (IFIMAC), Universidad Autónoma de Madrid, Madrid, Spain.*

[†]*These authors contributed equally to this work.*

* E-mail: gerrit.x.groenhof@jyu.fi

Contents

1 Molecular Dynamics Simulation Model **1**

1.1 Multiscale Tavis-Cummings Hamiltonian 1

1.2 One-dimensional Periodic Cavity 3

1.3 Mean-Field Non-Adiabatic Molecular Dynamics Simulations 5

1.4 Cavity Decay 6

2 Simulation details **8**

2.1 Rhodamine Model 8

2.1.1 Comparison of CIS/3-21G to higher levels of theory 9

2.1.2 Minimum Energy Conical Intersection 11

2.2 Tetracene Model 12

2.3 Methylene Blue Model 13

2.4 Molecular Dynamics of Rhodamine-Cavity Systems 14

2.5 Molecular Dynamics of Tetracene-Cavity Systems 18

2.6 Molecular Dynamics of Methylene-Blue Cavity System 19

2.7 Initial Conditions 20

2.7.1 On-resonant Excitation 20

2.7.2 Off-resonant Excitation 20

3 Simulation Analysis **22**

3.1 Contributions of Molecular and Cavity Mode Excitations to the Wavepacket 22

3.2 Monitoring Wavepacket Dynamics 22

3.3 Average Velocity 24

3.4 Photo-Absorption Spectra 24

3.5 Photo-Luminescence Spectra 25

4 Supplementary Results and Discussion **28**

4.1 Polariton Transport for Different Numbers of Molecules N 28
4.1.1 On-resonant Excitation 28
4.1.2 Off-resonant Excitation 34
4.2 Comparison between Uni- and Bi-directional Cavity Models 43
4.3 Simulations at 0 K 44
4.3.1 On-resonant Excitation 44
4.3.2 Off-resonant Excitation 47
4.4 Non-Adiabatic Transitions 49
4.5 Effect of Positional Disorder on Polariton Propagation 50
4.6 Effect of Initial Energy Disorder on Ballistic Transport 51
4.7 Effect of Rabi Splitting on Polariton Propagation 52
4.7.1 On-resonant Excitation 52
4.7.2 Off-resonant Excitation 54
4.8 Contribution of High-Energy Polariton States to the Wavepacket Propagation . . . 55
4.9 Polariton Propagation in Tetracene and Methylene Blue cavities 56
4.9.1 Tetracene 56
4.9.2 Methylene Blue 61

5 Animations **63**

6 Supplementary References **63**

1 Molecular Dynamics Simulation Model

1.1 Multiscale Tavis-Cummings Hamiltonian

Within the single-excitation subspace, probed experimentally under weak driving conditions, and employing the rotating wave approximation (RWA), valid for light-matter coupling strengths below 10% of the material excitation energy,[1] the interaction between N molecules and n_{mode} confined light modes in a one-dimensional (1D) Fabry-Pérot cavity (Supplementary Figure 1) is modelled with the multi-scale Molecular Dynamics (MD) extension of the traditional Tavis-Cummings model of quantum optics:[2–5]

$$\begin{aligned}\hat{H}^{\text{TC}} \quad &= \quad \sum_j^N h\nu_j(\mathbf{R}_j)\hat{\sigma}_j^+\hat{\sigma}_j^- + \sum_{k_z}^{n_{\text{mode}}} \hbar\omega_{\text{cav}}(k_z)\hat{a}_{k_z}^\dagger \hat{a}_{k_z} + \\ &\sum_j^N \sum_{k_z}^{n_{\text{mode}}} \hbar g_j(k_z)\left(\hat{\sigma}_j^+\hat{a}_{k_z}e^{ik_z z_j} + \hat{\sigma}_j^-\hat{a}_{k_z}^\dagger e^{-ik_z z_j}\right) + \\ &\sum_i^N V_{\text{S}_0}^{\text{mol}}(\mathbf{R}_i)\end{aligned} \tag{1}$$

Here, $\hat{\sigma}_j^+$ ($\hat{\sigma}_j^-$) is the operator that excites (de-excites) molecule j from the electronic ground (excited) state $|\text{S}_0^j(\mathbf{R}_j)\rangle$ ($|\text{S}_1^j(\mathbf{R}_j)\rangle$) to the electronic excited (ground) state $|\text{S}_1^j(\mathbf{R}_j)\rangle$ ($|\text{S}_0^j(\mathbf{R}_j)\rangle$); $\mathbf{R}_j$ is the vector of the Cartesian coordinates of all atoms in molecule j, centered at z_j; $\hat{a}_{k_z}$ ($\hat{a}_{k_z}^\dagger$) is the annihilation (creation) operator of an excitation of a cavity mode with wave-vector k_z; $h\nu_j(\mathbf{R}_j)$ is the excitation energy of molecule j, defined as:

$$h\nu_j(\mathbf{R}_j) = V_{\text{S}_1}^{\text{mol}}(\mathbf{R}_j) - V_{\text{S}_0}^{\text{mol}}(\mathbf{R}_j) \tag{2}$$

with $V_{\text{S}_0}^{\text{mol}}(\mathbf{R}_j)$ and $V_{\text{S}_1}^{\text{mol}}(\mathbf{R}_j)$ the adiabatic potential energy surfaces (PESs) of molecule j in the electronic ground (S_0) and excited (S_1) state, respectively. The last term in Equation 1 is the total potential energy of the system in the absolute ground state (*i.e.*, with no excitations in neither the molecules nor the cavity modes), defined as the sum of the ground-state potential energies of all

molecules in the cavity. The $V_{\mathrm{S}_0}^{\mathrm{mol}}(\mathbf{R}_j)$ and $V_{\mathrm{S}_1}^{\mathrm{mol}}(\mathbf{R}_j)$ adiabatic PESs are modelled at the hybrid quantum mechanics / molecular mechanics (QM/MM) level of theory.[6,7]

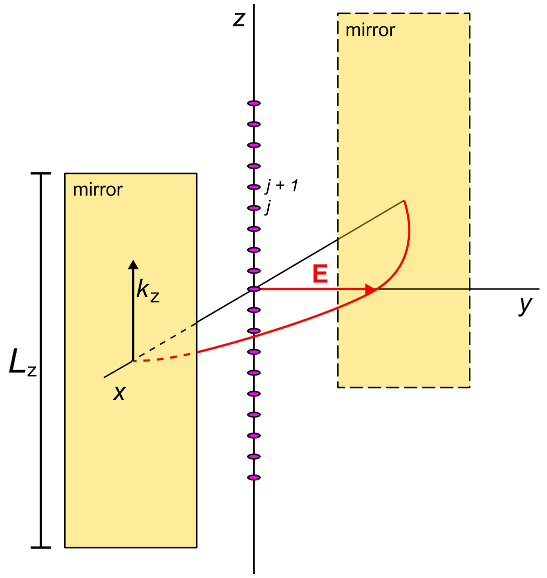

Supplementary Figure 1: One-dimensional (1D) Fabry-Pérot micro-cavity model. Two reflecting mirrors located at $-\frac{1}{2}x$ and $\frac{1}{2}x$, confine light modes along this direction, while free propagation along the z direction is possible for plane waves with in-plane momentum k_z and energy $\hbar\omega_{\mathrm{cav}}(k_z)$. The vacuum field vector (red) points along the y-axis, reaching a maximum amplitude at $x = 0$ where the N molecules (magenta ellipses) are placed, distributed along the z-axis at positions z_j with $1 \leq j \leq N$.

The third term in Equation 1 describes the light-matter interaction within the dipolar approximation through $g_j(k_z)$:

$$g_j(k_z) = -\boldsymbol{\mu}_j^{\mathrm{TDM}}(\mathbf{R}_j) \cdot \mathbf{u}_{\mathrm{cav}} \sqrt{\frac{\hbar\omega_{\mathrm{cav}}(k_z)}{2\epsilon_0 V_{\mathrm{cav}}}} \tag{3}$$

where $\boldsymbol{\mu}_j^{\mathrm{TDM}}(\mathbf{R}_j)$ is the transition dipole moment of molecule j that depends on the molecular geometry ($\mathbf{R}_j$); $\mathbf{u}_{\mathrm{cav}}$ the unit vector in the direction of the electric component of cavity vacuum field (*i.e.*, $|\mathbf{E}| = \sqrt{\hbar\omega_{\mathrm{cav}}(k_z)/2\epsilon_0 V_{\mathrm{cav}}}$), here along the y-direction (Supplementary Figure 1); ϵ_0 the vacuum permittivity; and V_{cav} the cavity mode volume.

1.2 One-dimensional Periodic Cavity

Following Michetti and La Rocca,[4] we impose periodic boundary conditions in the z-direction of our cavity, and thus restrict the wave vectors, k_z, to discrete values: $k_{z,p} = 2\pi p/L_z$ with $p \in \mathbb{Z}$ and L_z the length of the 1D cavity. With this approximation the molecular Tavis-Cummings Hamiltonian in Equation 1 can be represented as an $(N + n_{\text{mode}})$ by $(N + n_{\text{mode}})$ matrix with four blocks:[5]

$$\mathbf{H}^{\text{TC}} = \begin{pmatrix} \mathbf{H}^{\text{mol}} & \mathbf{H}^{\text{int}} \\ \mathbf{H}^{\text{int}\dagger} & \mathbf{H}^{\text{cav}} \end{pmatrix} \tag{4}$$

We compute the elements of this matrix in the product basis of adiabatic molecular states times cavity mode excitations:

$$\begin{aligned} |\phi_j\rangle &= \hat{\sigma}_j^+ |\text{S}_0^1\text{S}_0^2..\text{S}_0^{N-1}\text{S}_0^N\rangle \otimes |00..0\rangle \\ &= \hat{\sigma}_j^+ |\Pi_i^N \text{S}_0^i\rangle \otimes |\Pi_k^{n_{\text{mode}}} 0_k\rangle \\ &= \hat{\sigma}_j^+ |\phi_0\rangle \end{aligned} \tag{5}$$

for $1 \leq j \leq N$, and

$$\begin{aligned} |\phi_{j>N}\rangle &= \hat{a}_{j-N}^\dagger |\text{S}_0^1\text{S}_0^2..\text{S}_0^{N-1}\text{S}_0^N\rangle \otimes |00..0\rangle \\ &= \hat{a}_{j-N}^\dagger |\Pi_i^N \text{S}_0^i\rangle \otimes |\Pi_k^{n_{\text{mode}}} 0_k\rangle \\ &= \hat{a}_{j-N}^\dagger |\phi_0\rangle \end{aligned} \tag{6}$$

for $N < j \leq N + n_{\text{mode}}$. In these expressions $|00..0\rangle$ indicates that the Fock states for all n_{mode} cavity modes are empty. The basis state $|\phi_0\rangle$ is the ground state of the molecule-cavity system with

no excitations of neither the molecules nor cavity modes:

$$|\phi_0\rangle = |\mathrm{S}_0^1\mathrm{S}_0^2..\mathrm{S}_0^{N-1}\mathrm{S}_0^N\rangle \otimes |00..0\rangle = |\Pi_i^N \mathrm{S}_0^i\rangle \otimes |\Pi_k^{n_{\mathrm{mode}}} 0_k\rangle \tag{7}$$

The upper left block, $\mathbf{H}^{\mathrm{mol}}$, is an $N \times N$ matrix containing the single-photon excitations of the molecules. Because we neglect direct excitonic interactions between molecules, this block is diagonal, with elements labeled by the molecule indices j:

$$H_{j,j}^{\mathrm{mol}} = \langle\phi_0|\hat{\sigma}_j \hat{H}^{\mathrm{TC}} \hat{\sigma}_j^+|\phi_0\rangle \tag{8}$$

for $1 \leq j \leq N$. Each matrix element of $\mathbf{H}^{\mathrm{mol}}$ thus represents the potential energy of a molecule, j, in the electronic excited state $|\mathrm{S}_1^j(\mathbf{R}_j)\rangle$ while all other molecules, $i \neq j$, are in the electronic ground state $|\mathrm{S}_0^i(\mathbf{R}_i)\rangle$:

$$H_{j,j}^{\mathrm{mol}} = V_{\mathrm{S}_1}^{\mathrm{mol}}(\mathbf{R}_j) + \sum_{i \neq j}^{N} V_{\mathrm{S}_0}^{\mathrm{mol}}(\mathbf{R}_i) \tag{9}$$

The lower right block, $\mathbf{H}^{\mathrm{cav}}$, is an $n_{\mathrm{mode}} \times n_{\mathrm{mode}}$ matrix (with $n_{\mathrm{mode}} = n_{\mathrm{max}} - n_{\mathrm{min}} + 1$) containing the single-photon excitations of the cavity modes, and is also diagonal:

$$H_{p,p}^{\mathrm{cav}} = \langle\phi_0|\hat{a}_p \hat{H}^{\mathrm{TC}} \hat{a}_p^\dagger|\phi_0\rangle \tag{10}$$

for $n_{\mathrm{min}} \leq p \leq n_{\mathrm{max}}$. Here, $\hat{a}_p^\dagger$ excites cavity mode p with wave-vector $k_{z,p} = 2\pi p/L_z$. In these matrix elements, all molecules are in the electronic ground state (S_0). The energy is therefore the sum of the cavity energy at $k_{z,p}$, and the molecular ground state energies:

$$H_{p,p}^{\mathrm{cav}} = \hbar\omega_{\mathrm{cav}}(2\pi p/L_z) + \sum_{j}^{N} V_{\mathrm{S}_0}^{\mathrm{mol}}(\mathbf{R}_j) \tag{11}$$

where, $\omega_{\mathrm{cav}}(k_{z,p})$ is the cavity dispersion (dashed curve in Figure 1b, main text):

$$\omega_{\mathrm{cav}}(k_{z,p}) = \sqrt{\omega_0^2 + c^2 k_{z,p}^2/n^2} \tag{12}$$

with $\hbar\omega_0$ the energy at $k_0 = 0$, n the refractive index of the medium and c the speed of light in vacuum.

The two $N \times n_{\text{mode}}$ off-diagonal blocks $\mathbf{H}^{\text{int}}$ and $\mathbf{H}^{\text{int}\dagger}$ in the multi-mode Tavis-Cummings Hamiltonian (Equation 4) model the light-matter interactions between the molecules and the cavity modes. These matrix elements are approximated as the overlap between the transition dipole moment of molecule j and the transverse electric field of the cavity mode p at the geometric center z_j of that molecule:

$$\begin{aligned} H_{j,p}^{\text{int}} &= -\boldsymbol{\mu}_j^{\text{TDM}}(\mathbf{R}_j) \cdot \mathbf{u}_{\text{cav}} \sqrt{\frac{\hbar\omega_{\text{cav}}(2\pi p/L_z)}{2\epsilon_0 V_{\text{cav}}}} \langle\phi_0|\hat{\sigma}_j\hat{\sigma}_j^+\hat{a}_p e^{i2\pi p z_j/L_z}\hat{a}_p^\dagger|\phi_0\rangle \\ &= -\boldsymbol{\mu}_j^{\text{TDM}}(\mathbf{R}_j) \cdot \mathbf{u}_{\text{cav}} \sqrt{\frac{\hbar\omega_{\text{cav}}(2\pi p/L_z)}{2\epsilon_0 V_{\text{cav}}}} e^{i2\pi p z_j/L_z} \end{aligned} \tag{13}$$

for $1 \leq j \leq N$ and $n_{\text{min}} \leq p \leq n_{\text{max}}$.

Diagonalization of the multi-scale Tavis-Cummings Hamiltonian in Equation 4 yields the $N + n_{\text{mode}}$ hybrid light-matter states $|\psi^m\rangle$:[4,8]

$$|\psi^m\rangle = \left(\sum_j^N \beta_j^m \hat{\sigma}_j^+ + \sum_p^{n_{\text{mode}}} \alpha_p^m \hat{a}_p^\dagger\right)|\phi_0\rangle \tag{14}$$

with eigenenergies E_m. The β_j^m and α_p^m expansion coefficients reflect the contribution of the molecular excitons ($|\mathrm{S}_1^j(\mathbf{R}_j)\rangle$) and the cavity mode excitations ($|1_p\rangle$) to polariton $|\psi^m\rangle$.

1.3 Mean-Field Non-Adiabatic Molecular Dynamics Simulations

Semi-classical Molecular Dynamics (MD) trajectories of all molecules are computed by numerically integrating Newton's equations of motion. The multi-mode Tavis-Cummings Hamiltonian (Equation 4) is diagonalized at each time-step to obtain the $N + n_{\text{mode}}$ (adiabatic) polaritonic eigenstates $|\psi^m\rangle$ and energies E^m. The *total* polaritonic wavefunction $|\Psi(t)\rangle$ is coherently propagated along with the classical degrees of freedom of the N molecules as a time-dependent superposition of the

$N + n_{\text{mode}}$ time-independent adiabatic polaritonic states:

$$|\Psi(t)\rangle = \sum_{m}^{N+n_{\text{mode}}} c_m(t)|\psi^m\rangle \tag{15}$$

where $c_m(t)$ are the time-dependent expansion coefficients of the time-independent polaritonic basis functions $|\psi^m\rangle$ defined in Equation 14. A unitary propagator in the *local* diabatic basis is used to integrate these coefficients,[9] while the nuclear degrees of freedom of the N molecules evolve on the mean-field PES:

$$V(\mathbf{R}) = \langle\Psi(t)|\hat{H}^{\text{TC}}|\Psi(t)\rangle \tag{16}$$

Population transfers between polaritonic states $|\psi^m\rangle$ and $|\psi^l\rangle$ are driven by the non-adiabatic coupling, or vibronic coupling $V_{m,l}^{\text{NAC}}$.[10–12] $V_{m,l}^{\text{NAC}}$ depends on the overlap between the non-adiabatic coupling vector $\mathbf{d}_{m.l}$ and the velocities of the atoms $\dot{\mathbf{R}}$:[13]

$$V_{m,l}^{\text{NAC}} = \mathbf{d}_{m,l} \cdot \dot{\mathbf{R}} \tag{17}$$

where $\dot{\mathbf{R}}$ is a vector containing the velocities of all atoms in the system, and the non-adiabatic coupling vector $\mathbf{d}_{m,l}$ is given by:

$$\mathbf{d}_{m,l} = \frac{\langle\psi^m|\nabla_{\mathbf{R}}\hat{H}^{\text{TC}}|\psi^l\rangle}{E_l - E_m} \tag{18}$$

where E_m and E_l are the eigenvalues associated with $|\psi^m\rangle$ and $|\psi^l\rangle$, respectively. The direction of $\mathbf{d}_{m,l}$ determines the direction of population transfer, while its magnitude determines the amount of population that is transferred.

1.4 Cavity Decay

Radiative loss through the cavity mirrors is modeled as a first-order decay process into the overall ground state of the system (*i.e.*, no excitation in neither the molecules nor the cavity modes).[14]

Assuming that the intrinsic decay rates γ_{cav} are the same for all modes, the total loss rate is calculated as the product of γ_{cav} and the total photonic weight, $\sum_p^{n_{\text{mode}}} |\alpha_p^m|^2$, of state $|\psi^m\rangle$. Thus, after an MD step Δt, $\rho_m(t) = |c_m(t)|^2$ becomes:

$$\rho_m(t + \Delta t) = \rho_m(t) \exp\left[-\gamma_{\text{cav}} \sum_p^{n_{\text{mode}}} |\alpha_p^m(t)|^2 \Delta t\right] \tag{19}$$

Since $\rho_m = (\Re[c_m])^2 + (\Im[c_m])^2$, changes in the real and imaginary parts of the (complex) expansion coefficients $c_m(t)$, due to spontaneous photonic loss in a low-Q cavity, are:

$$\Re[c_m(t + \Delta t)] = \Re[c_m(t)] \exp\left[-\frac{1}{2}\gamma_{\text{cav}} \sum_p^{n_{\text{mode}}} |\alpha_p^m(t)|^2 \Delta t\right]$$

$$\Im[c_m(t + \Delta t)] = \Im[c_m(t)] \exp\left[-\frac{1}{2}\gamma_{\text{cav}} \sum_p^{n_{\text{mode}}} |\alpha_p^m(t)|^2 \Delta t\right]$$

Simultaneously, the population of the zero-excitation subspace, or ground state, $\rho_0(t+\Delta t)$, increases as:

$$\rho_0(t + \Delta t) = \rho_0(t) + \sum_m \rho_m(t) \left(1 - \exp\left[-\gamma_{\text{cav}} \sum_p^{n_{\text{mode}}} |\alpha_p^m(t)|^2 \Delta t\right]\right) \tag{20}$$

2 Simulation details

2.1 Rhodamine Model

Rhodamine molecules, one of which is shown schematically in Supplementary Figure 2a, were modelled with the Amber03 force field,[15] using the parameters provided by Luk *et al.*[14] After a geometry optimization at the force field level, the molecule was placed at the center of a rectangular box and 3,684 TIP3P water molecules,[16] were added. The simulation box, which contained 11,089 atoms, was equilibrated for 2 ns with harmonic restraints on the heavy atoms of Rhodamine (force constant 1000 $kJmol^{-1}nm^{-1}$). Subsequently, a 200 ns classical MD trajectory was computed at constant temperature (300 K) using a stochastic dynamics integrator with a friction coefficient of 0.1 ps^{-1}. The pressure was kept constant at 1 bar using the Berendsen isotropic pressure coupling algorithm[17] with a time constant of 1 ps. The LINCS algorithm was used to constrain bond lengths,[18] while SETTLE was applied to constrain the internal degrees of freedom of water molecules,[19] enabling a time step of 2 fs in the classical MD simulations. A 1.0 nm cut-off was used for Van der Waals' interactions, which were modeled with Lennard-Jones potentials. Coulomb interactions were computed with the smooth particle mesh Ewald method,[20] using a 1.0 nm real space cut-off and a grid spacing of 0.12 nm. The relative tolerance at the real space cut-off was set to 10^{-5}.

From the second half of the 200 ns MD trajectory, snapshots were extracted and subjected to further equilibration for 10 ps at the QM/MM level. The time step was reduced to 1 fs. As in previous work,[5,14,21] the fused ring system was included in the QM region and described at the RHF/3-21G level, while the rest of the molecule as well as the water solvent were modelled with the Amber03 force field[15] and TIP3P water model,[16] respectively (Supplementary Figure 2). The bond connecting the QM and MM subsystems was replaced by a constraint and the QM part was capped with a hydrogen atom. The force on the cap atom was distributed over the two atoms of the bond. The QM system experienced the Coulomb field of all MM atoms within a 1.6 nm cut-off sphere and Lennard-Jones interactions between MM and QM atoms were added. The singlet electronic excited

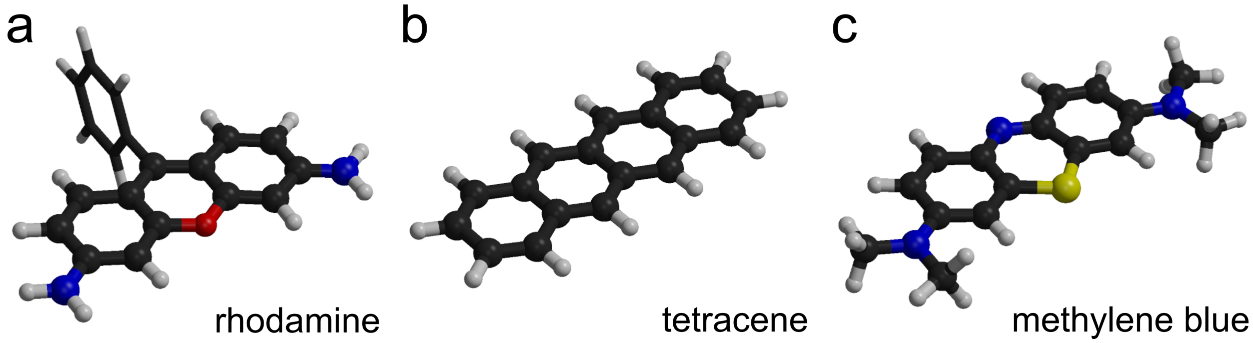

Supplementary Figure 2: Panel **a**: Rhodamine model used in our simulations. The QM subsystem, shown in ball-and-stick representation, is described at the HF/3-21G level of theory in the electronic ground state (S_0), and at the CIS/3-21G level of theory in the first singlet excited state (S_1). The MM subsystem, consisting of the atoms shown in stick representation, and the water molecules (not shown), are modelled with the Amber03 force field. Panel **b**: Tetracene model. All atoms are included in the QM subsystem, modelled at the RHF/3-21G level of the theory in S_0 and at the CIS/3-21G level of theory in S_1. The cyclohexane solvent (not shown) is included in the MM subsystem and described with the Gromos96-54a7 force field. Panel **c**: Methylene Blue model. All atoms are included in the QM subsystem, modelled at the B97/3-21G level of the theory in S_0 and at the TDA-B97/3-21G level of theory in S_1. The water solvent (not shown) is included in the MM subsystem and described with the Amber03 force field.

state (S_1) was modeled with the Configuration Interaction method, truncated at single electron excitations, for the QM region (*i.e.,* CIS/3-21G//Amber03). At this level of QM/MM theory, the excitation energy is 4.18 eV [14]. The QM/MM simulations were performed with GROMACS version 4.5.3,[22] interfaced to TeraChem version 1.93.[23,24]

2.1.1 Comparison of CIS/3-21G to higher levels of theory

The level of theory used in this work is a necessary compromise between efficiency and accuracy. Because no experimental data is available for the Rhodamine model used here, we instead verified the validity of our model by recomputing the electronic energies along an excited-state molecular dynamics trajectory of a single Rhodamine molecule in water at higher levels of theory:

- Time-dependent density functional theory (TDA-DFT)[25] within the Tamm-Dancoff approximation[26] with the CAM-B3LYP functional,[27,28] in combination with the 6-31G(d) basis set;[29]

- Complete Active Space Self Consistent Field (CASSCF) with 12 electrons in 12 π orbitals,[30] averaged over the first two states (SA2) and expanded in a cc-pVDZ basis set;[31]

- Extended Multi-Configuration Quasi-Degenerate Perturbation Theory to second order (xMC-QDPT2),[32] based on the SA2-CASSCF(12,12)/cc-pVDZ reference.

The TD-DFT calculations were carried out with the Gaussian09 program,[33] while the CASSCF and xMCQDPT2 calculations were performed with Firefly.[34]

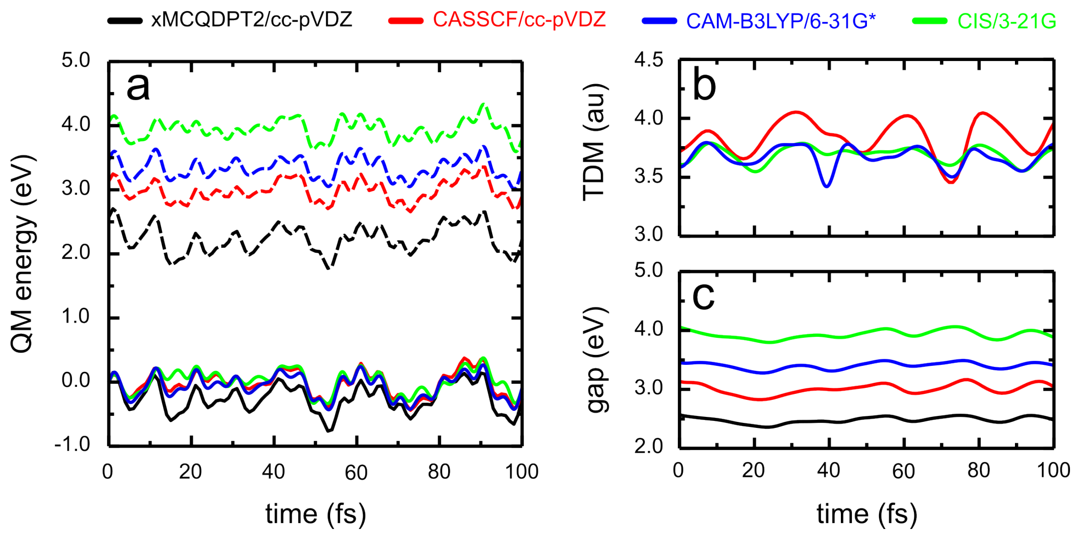

Supplementary Figure 3: Panel **a**: potential energies in the electronic ground (S_0, continuous lines) and excited state (S_1, dashed lines) as a function of time in an excited-state Molecular Dynamics (MD) trajectory of a single Rhodamine at the Configuration Interaction restricted to single excitations and the 3-21G basis set (CIS/3-21G, green) level of theory, re-evaluated at the Extended Multi-Configuration Quasi-Degenerate Perturbation Theory to second order, based on a two-state-averaged Complete Active Space Self Consistent Field reference and the correlation-consistent polarizable valence-only double-zeta basis set (xMCQDPT2//SA2-CASSCF(12,12)/cc-pVDZ, black), two-state-averaged Complete Active Space Self Consistent Field with the correlation-consistent polarizable valence-only double-zeta basis set (SA2-CASSCF(12,12)/cc-pVDZ, red), and time-dependent density functional theory within the Tamm-Dancoff approximation for the CAM-B3LYP functional and the 6-31G(d) basis set (TDA-CAM-B3LYP/6-31G(d), blue) levels of theory. Panel **b**: the transition dipole moment (TDM) in atomic units at the various levels of theory. Note that because the xMCQDPT2 method only corrects the energy, but not the wave function, the transition dipole moment is the same as that of the underlying CASSCF reference. Panel **c**: the energy gaps between the excited and ground state.

As shown in Supplementary Figure 3, the absolute excitation energy is overestimated at the CIS/3-21G level of theory with respect to the more accurate xMCQDPT2//SA2-CASSCF(12,12)/cc-

pVDZ approach. However, the positions of the minima and maxima coincide at all levels of theory. Therefore, we conclude that the topology of the PES is not very sensitive to the level of theory and that the nuclear dynamics will be sufficiently similar at all levels of theory.

Furthermore, the Stokes shift, which was shown to have an important effect on polariton relaxation,[35] was evaluated by first optimizing the molecule in the ground and excited state and then taking the difference between the vertical excitation energies at these minima. At the CIS/3-21G level of theory the Stokes shift of 0.12 meV is in somewhat better agreement with the xMCQDPT2//SA2-CASSCF(12,12)/cc-pVDZ value of 0.200 meV than SA2-CASSCF(12,12)/cc-pVDZ, which yields 338 meV, or TD-CAMB3LYP/6-31G(d), which yields 88 meV.

Because the polaritonic states are determined by the coupling between the cavity field and the molecular transition dipole moments, we also compared the transition dipole moment between the different levels of theory (Supplementary Figure 3b). As with the electronic energies, there are deviations, but the average transition dipole moments are within 0.1 au (0.25 Debeye).

Because we can compensate the overestimated excitation energy by adding an offset to the cavity dispersion in our simulations, we conclude that for the purposes of our study, the CIS/3-21G method provides an acceptable compromise between desired accuracy and computational efficiency for calculating trajectories of ensembles of Rhodamine molecules strongly coupled to the confined light modes of a Fabry-Pérot micro-cavity.

2.1.2 Minimum Energy Conical Intersection

There are three important points on the S_1 PES of the Rhodamine molecule: (i) the Frank-Condon (FC) vertical excitation point, (ii) the (local) minimum on the S_1 state and (iii) the minimum energy conical intersection (MECI) point between S_1 and S_0 states. We have optimized these points at the unified SA2-CASSCF(12,12)/cc-pVDZ level of theory and compared their energies to understand whether on the timescales of our simulations radiationless deactivation of the Rhodamine molecule via the conical intersection could play a role in the transport process.

First, we optimized the ground state geometry of the QM subsystem at the PBE0/cc-pVDZ

level of theory. With this geometry, we evaluated the vertical excitation energy into the FC point at the SA2-CASSCF(12,12)/cc-pVDZ level of theory. Next, we optimized the geometry on the S_1 excited state PES at the same CASSCF level of theory. Finally, we optimized the MECI point with the Firefly[34] package at the same SA2-CASSCF(12,12)/cc-pVDZ level of theory. The convergence criterion for all optimizations was that the maximum Cartesian gradient component is below 0.0001 a.u.

Results of the optimizations are summarized in Supplementary Table 1, in which the first colomn contains the absolute S_1 energies in Hartrees, while the second colomn lists the relative energies with respect to the FC energy in kJmol^{-1}. Because the MECI point is more than 120 kJmol^{-1} higher in energy than FC point, it is extremely unlikely that the system would sample the MECI region and undergo a radiationless decay into the S_0 ground state on the time scale of our simulations. Instead, the most probably deactivation process for bare Rhodamine is fluorescence, in line with experiment.

Supplementary Table 1: Computed S_1 state energies for the model rhodamine molecule in a gas-phase at the SA2-CASSCF(12,12)/cc-pVDZ level of theory

Geometry	E(S_1), a.u.	ΔE(S_1), kJmol^{-1}
FC	-682.337367	0
S_1 minimum	-682.342427	-13.29
MECI	-682.289575	125.48

2.2 Tetracene Model

Tetracene (Supplementary Figure 2b) was placed at the center of a rectangular box that was filled with 265 cyclohexane molecules. Intermolecular interactions were modelled with the Gromos96-54a7 united-atom force field.[36] The simulation box, which contained 2,785 atoms, was equilibrated for 100 ns. During equilibration, the coordinates of the Tetracene atoms were kept fixed. The LINCS algorithm was used to constrain bond lengths in cyclohexane,[18] enabling a time step of 2 fs. Temperature and pressure were maintained at 300 K and 1 atmosphere by means of weak-coupling to an external bath ($\tau_T = 0.1$ ps, $\tau_p = 1$ ps).[17] Because cyclohexane atoms are uncharged, the interactions between Tetracene and cyclohexane were modeled with Lennard-Jones potentials only.

The Lennard-Jones potential was truncated at 1.4 nm.

Snapshots were extracted from the equilibration trajectory and further equilibrated at the QM/MM level with a time step of 1 fs. In all QM/MM simulations, Tetracene was modelled at the restricted Hartree-Fock level of theory in combination with a 3-21G basis set and mechanically embedded into the MM region, which consisted of the cyclohexane solvent described with the Gromos96-54A7 united-atom force field.[36]. The Lennard-Jones interactions between the QM and MM subsystems were truncated at 1.4 nm. As in previous work,[37], we used the Configuration Interaction truncated at the single excitations (CIS), in combination with the 3-21G basis set,[29] to model the singlet excited electronic state of Tetracene. At this level of theory the absorption maximum is 3.87 eV, which is overestimated with respect to experiments due to the lack of electron-electron correlation in the CIS method and the limited flexibility of the 3-21G basis set. The QM/MM simulations were performed with GROMACS version 4.5.3,[22] interfaced to TeraChem version 1.93.[23,24]

2.3 Methylene Blue Model

Methylene Blue (Supplementary Figure 2c) was placed at the center of a rectangular box that was filled with 2,031 TIP3P water molecules.[16] Non-covalent interactions between Methylene Blue and water were modelled with the Amber03 force field.[15] A 1.0 nm cut-off was used for the Van der Waals' interactions, which were modeled with Lennard-Jones potentials, while the Coulomb interactions were computed with the smooth particle mesh Ewald method,[20] using a 1.0 nm real space cut-off and a grid spacing of 0.12 nm and a relative tolerance at the real space cut-off of 10^{-5}. The simulation box, containing 6,131 atoms, was equilibrated for 1 ns at the force field level of theory. During this equilibration, the coordinates of the Methylene atoms were kept fixed. The temperature was kept at 300 K with the v-rescale thermostat,[38] while the pressure was kept constant at 1 atmosphere using the Berendsen isotropic pressure coupling algorithm[17] with a time constant of 1 ps. The SETTLE algorithm was applied to constrain the internal degrees of freedom of water molecules,[19] enabling a time step of 2 fs in the classical MD simulations.

After the equilibration at the force field (MM) level, the system was further equilibrated for 10 ps at the QM/MM level. The time step was reduced to 1 fs. The Methylene Blue molecule was modelled with density functional theory, using the B97 functional,[39] in combination with the 3-21G basis set.[29] The water solvent was modelled with the TIP3P force field.[16] The QM system experienced the Coulomb field of all MM atoms within a 1.0 nm cut-off sphere and Lennard-Jones interactions between MM and QM atoms were added. The singlet electronic excited state (S_1) was modeled with time-dependent density functional theory,[25] using the B97 functional in combination with the 3-21G basis set for the QM region (*i.e.,* TD-B97/3-21G),[29]. At this level of QM/MM theory, the excitation energy is 2.5 eV. The overestimation of the vertical excitation energy is due to the limited accuracy of the employed level of theory, but was compensated by adding an off-set to the cavity resonance energy. The QM/MM simulations were performed with GROMACS version 4.5.3,[22] interfaced to TeraChem version 1.93.[23,24]

2.4 Molecular Dynamics of Rhodamine-Cavity Systems

After equilibration at the QM/MM level, the Rhodamine molecules in solution were placed with equal inter-molecular distances on the z-axis of a 1D,[4] 50 µm long, optical Fabry-Pérot micro-cavity (Supplementary Figure 1). The dispersion of this cavity was modelled with 160 discrete modes (*i.e.*, $k_{z,p} = 2\pi p/L_z$ with $0 \leq p \leq 159$ and $L_z = 50$ µm). The microcavity was red-detuned with respect to the Rhodamine absorption maximum, which is 4.18 eV at the CIS/3-21G//Amber03 level of theory used in this work, such that the energy of the cavity photon at zero incidence angle ($k_0 = 0$) was $\hbar\omega_0 = 3.81$ eV, corresponding to a distance of $L_x = 0.163$ µm between the mirrors (Supplementary Figure 1). To maximize the collective light-matter coupling strength, the transition dipole moments of the Rhodamine molecules were aligned to the vacuum field at the start of the simulation. Both lossless ($\hbar\gamma_{\text{cav}} = 0$ eV or $\gamma_{\text{cav}} = 0$ ps^{-1}, Equation 19) and lossy ($\hbar\gamma_{\text{cav}} = 0.04$ eV or $\gamma_{\text{cav}} = 66.7$ ps^{-1}) mirrors were considered such that the lifetime, τ_{cav}, of the lossy cavity is comparable to the 2-14 fs lifetimes of metallic Fabry-Pérot cavities used in experiments of strong coupling with organic molecules.[40–42] With a lifetime of 15 fs and a resonance at 3.81 eV, the

Quality-factor, defined as Q $= \omega_{\rm cav}\tau_{\rm cav}$, for the lossy cavity would be 87. We note, however, that this Q-factor is artificially high, because to compensate for the overestimated excitation energies at the CIS/3-21G level of *ab initio* theory (section 2.1.1), we added an offset to the cavity dispersion. Under the rotation wave approximation (RWA) and within the single-excitation subspace, valid under the weak driving conditions that are typically employed in experiments on exciton transport in organic micro-cavities, the absolute energy scale is irrelevant and therefore adding such offset has no direct impact on the dynamics of the molecule-cavity system.

We assessed the robustness of our approach with respect to the ensemble size by investigating the effect of varying the number of molecules N. In a first set of simulations the same starting coordinates were used for all Rhodamines, but different initial velocities were selected randomly from a Maxwell-Boltzmann distribution at 300 K. To vary N while keeping the Rabi splitting the same ($\sim$ 325 meV), we scaled the cavity volume by the number of Rhodamine molecules (Equation 3), yielding cavity vacuum fields of $\mathbf{E}_y = 0.0001$ a.u. (0.514 MVcm^{-1}) for 256 molecules, $\mathbf{E}_y = 0.0000707107$ a.u. (0.364 MVcm^{-1}) for 512 molecules and $\mathbf{E}_y = 0.00005$ a.u (0.257 MVcm^{-1}) for 1024 molecules. With identical Rabi splitting energies, the polariton dispersion, and hence also the polariton group velocities, defined as $v_{\rm gr}^{\rm LP}(k_{z,p}) = \frac{\partial\omega(k_{z,p})}{\partial k_{z,p}}$, are the same.

To also explore the effect of initial energy disorder, the starting coordinates of Rhodamines were varied in a second set of simulations. For these simulations, the initial conformations and velocities were taken from the QM/MM equilibrium trajectories.

To investigate the effect of the Rabi splitting on polariton transport, we performed a third set of simulations with 1024 molecules at multiple cavity vacuum field strengths: $\mathbf{E}_y = 0.000025$, 0.000037 and 0.00005 a.u., yielding Rabi splittings of $\hbar\Omega^{\rm Rabi} = 159$, 240, and 325 meV. In these simulations, the same starting coordinates were used for all Rhodamines, but with different initial velocities, selected randomly from a Maxwell-Boltzmann distribution at 300 K. For each Rabi splitting, the simulations were started with the same random velocities.

For systems without disorder, bright states and dark states are easily distinguishable because dark states lack contributions from cavity mode excitations. In contrast, in the presence of disorder due to

molecules adopting different conformations in the simulations (and in reality), the cavity modes are smeared out over many states.[43] Nevertheless, because the cavity modes are not distributed evenly over all states, the upper (UP) and lower (LP) polaritonic branches remain visible, even if the number of eigenstates that make up these branches exceeds the number of cavity modes. Therefore, to have sufficient dark states, loosely defined as states with a negligible cavity mode contribution, the ratio between the number of dark (N_{dark}) and polaritonic states (N_{pol}) must be larger than zero. While this is the case when there are 1024 ($N_{\text{dark}}/N_{\text{pol}} = 2.7$), 512 ($N_{\text{dark}}/N_{\text{pol}} = 1.1$), or 256 molecules ($N_{\text{dark}}/N_{\text{pol}} = 0.3$) in the cavity, the smallest cavity systems with 160 molecules ($N_{\text{dark}}/N_{\text{pol}} = 0$), has no real dark state manifold, which, as we show below, affects the propagation mechanism.

Here, we determined these ratios for an ideal system without disorder, in which all molecules are identical and the polaritonic and dark states can be easily identified. Furthermore, because in this ideal scenario bright polaritons and degenerate dark states form irrespective of the coupling strength (provided it is not zero), their ratio is independent of the Rabi splitting and only depends on N as $N_{\text{dark}}/N_{\text{pol}} = (N - n_{\text{modes}})/2n_{\text{modes}}$.

In contrast, when there is thermal disorder, as in our MD simulations, the cavity modes are smeared-out over many eigenstates.[21,43,44] In this situation, there is no longer a clear distinction between dark and polaritonic states. While we could in principle apply a numerical criterion and consider a state $|\psi^m\rangle$ polaritonic if the total cavity mode contribution to that state exceeds an arbitrary threshold, ϵ (*i.e.*, $\sum_p |\alpha_p^m|^2 > \epsilon$, Equation 14),[5,21] such an approach would make the ratio dependent on the choice of that threshold.

Nevertheless, as shown Supplementary Figure 4**a**, calculating the ratio at constant Rabi splitting (325 meV) for various thresholds, *i.e.*, $\epsilon = 0.01$, 0.02 and 0.05, with energies and transition dipole moments extracted from the QM/MM equilibrium trajectory at 300 K, suggests that irrespective of the value for the threshold parameter, the ratio between dark and polaritonic states scales approximately linearly with N, but with a slope that depends on the threshold.

Furthermore, the extent to which the cavity mode excitations are smeared-out over the states not only depends on how large the disorder is, but also on the collective coupling strength, or

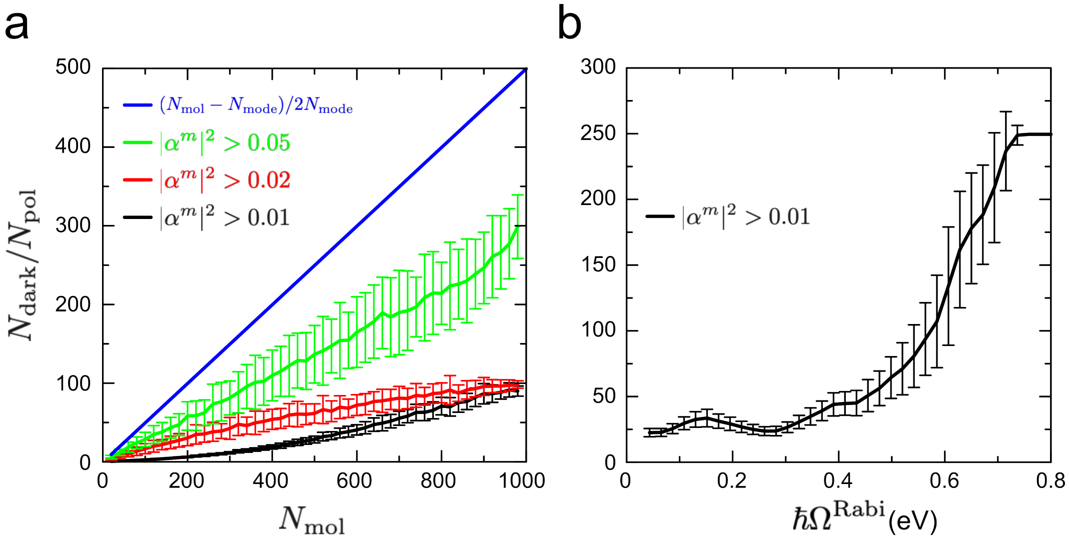

Supplementary Figure 4: In panel **a** the ratio between dark and polaritonic states at a constant Rabi splitting (325 meV) is plotted as a function of the number of molecules in a disordered ensemble collectively coupled to a single mode cavity that is resonant with the molecular excitation energy (4.18 ev), for various values of the threshold parameter ϵ (*i.e.*, $|\alpha^m|^2 > \epsilon$).[21] The ratio for a system without disorder is shown in blue. The plots show the ratios averaged over 1000 ensembles of N_{mol} molecules randomly selected from the QM/MM equilibrium trajectory at 300 K. The error bars represent the standard deviation. Panel **b** shows the ratio as a function of Rabi splitting for an ensemble with 500 molecules and a threshold of $\epsilon = 0.01$. For each Rabi splitting, the ratio shown is the average over 1000 ensembles of 512 molecules randomly selected from the QM/MM equilibrium trajectory at 300 K. The error bars represent the standard deviation.

equivalently, the Rabi splitting.[21,43] Indeed, as shown in Supplementary Figure 4**b**, calculating the ratios at different coupling strengths, but for fixed $N = 500$ and a numerical threshold of $\epsilon = 0.01$, suggest that at the Rabi splitting in our simulations (325 meV), the ratio is significantly smaller with disorder than without disorder. However, because we adjusted the vacuum field strength to keep the Rabi splitting the same for all systems, and the ratio is proportional to N at constant Rabi splitting (Supplementary Figure 4**a**), the dependence on the Rabi splitting does not influence the comparison between the different ensembles. Nevertheless, to avoid complications associated with choosing an arbitrary threshold, we estimated the ratios for systems with different numbers of molecules, based on the ideal situation without disorder, in which the distinction between polaritonic and dark states is not only unambiguous but also independent of the Rabi splitting and the molecular properties.

We used a leap-frog Verlet algorithm with a 0.1 fs time step (0.5 fs, for simulations at different

Rabi splittings) to perform the integration of the nuclear dynamics, while the unitary propagator in the local diabatic basis was used to propagate the polaritonic degrees of freedom.[9] To avoid self-interference of the wavepacket, the simulations were terminated far before a wavepacket could reach the periodic boundary of the 1D cavity at $z_{N+1} = z_1$. All results were obtained as averages of at least two simulations. For all cavity simulations we used Gromacs 4.5.3,[22] in which the multi-mode Tavis-Cummings QM/MM model was implemented,[5] in combination with Gaussian16.[45]

2.5 Molecular Dynamics of Tetracene-Cavity Systems

After equilibration at the QM/MM level 1024 Tetracene molecules in cyclohexane were placed with equal inter-molecular distances on the z-axis of a 1D,[4] 50 μm long, optical Fabry-Pérot micro-cavity (Supplementary Figure 1). The dispersion of this cavity was modelled with 160 discrete modes (*i.e.*, $k_{z,p} = 2\pi p/L_z$ with $0 \leq p \leq 159$ and $L_z = 50$ μm). The microcavity was red-detuned with respect to the Tetracene absorption maximum, which is 3.87 eV at the CIS/3-21G//Gromos96-54a7 level of theory used in this work, such that the energy of the cavity photon at zero incidence angle ($k_0 = 0$) was $\hbar\omega_0 = 3.55$ eV. To maximize the collective light-matter coupling strength, the transition dipole moments of Tetracene molecules were aligned to the vacuum field at the start of the simulation. With a vacuum field strength of $\mathbf{E}_y = 0.0001$ a.u. (0.51 MVcm^{-1}) this configuration yields a Rabi splitting of $\hbar\Omega^{\text{Rabi}} = 301$ meV. Simulations were performed for both lossless ($\hbar\gamma_{\text{cav}} = 0$ ps^{-1}) and a lossy cavities ($\hbar\gamma_{\text{cav}} = 66.7$ ps^{-1}) such that the lifetime, τ_{cav}, of the lossy cavity is comparable to the 2-14 fs lifetimes of metallic Fabry-Pérot cavities used in experiments of strong coupling with organic molecules.[40–42] .

A leap-frog Verlet algorithm with a 0.1 fs time step was used to integrate the nuclear dynamics, while the unitary propagator in the local diabatic basis was used to propagate the polaritonic degrees of freedom.[9] To avoid self-interference of the wavepacket, the simulation was stopped before a wavepacket could reach the periodic boundary of the 1D cavity at $z_{N+1} = z_1$. Results were obtained as averages of three independent simulations that were performed with Gromacs 4.5.3,[22] in which the multi-mode Tavis-Cummings QM/MM model was implemented,[5] in combination with

Gaussian16.[45]

2.6 Molecular Dynamics of Methylene-Blue Cavity System

After equilibration at the QM/MM level 1024 Methylene Blue molecules in solution were placed with equal inter-molecular distances on the z-axis of a 1D,[4] 50 μm long, optical Fabry-Pérot micro-cavity (Supplementary Figure 1). The dispersion of this cavity was modelled with 160 discrete modes (*i.e.*, $k_{z,p} = 2\pi p/L_z$ with $0 \leq p \leq 159$ and $L_z = 50$ μm). The micro-cavity was red-detuned with respect to the Methylene Blue absorption maximum, which is 2.5 eV at the TD-B97/3-21G//Amber03 level of theory used in this work, such that the energy of the cavity photon at zero incidence angle ($k_0 = 0$) was $\hbar\omega_0 = 1.9$ eV. To maximize the collective light-matter coupling strength, the transition dipole moments of the Methylene Blue molecules were aligned to the vacuum field at the start of the simulation. With a vacuum field strength of $\mathbf{E}_y = 0.000025$ a.u. (0.128 MVcm^{-1}) this configuration yields a Rabi splitting of $\hbar\Omega^{\text{Rabi}} = 175$ meV. Because the purpose of this additional simulation was to confirm the general transport mechanism, rather than explore the effect of molecular or cavity parameters, we only considered an ideal lossless cavity ($\hbar\gamma_{\text{cav}} = 0$ eV or $\gamma_{\text{cav}} = 0$ ps^{-1}, Equation 19).

A leap-frog Verlet algorithm with a 0.5 fs time step was used to integrate the nuclear dynamics, while the unitary propagator in the local diabatic basis was used to propagate the polaritonic degrees of freedom.[9] To avoid self-interference of the wave packet, the simulation was ended before a wavepacket could reach the periodic boundary of the 1D cavity at $z_{N+1} = z_1$. The simulation was performed with Gromacs 4.5.3,[22] in which the multi-mode Tavis-Cummings QM/MM model was implemented,[5] in combination with Gaussian16.[45]

2.7 Initial Conditions

2.7.1 On-resonant Excitation

Following Agranovich and Garstein,[8] on-resonant excitation of a wavepacket of LP states was achieved by assigning to the expansion coefficients $c_m(t = 0)$ values of a Gaussian distribution centered at k-vector k_c = $55\frac{2\pi}{L_z}$ = 6.91 μm^{-1} corresponding to the maximum group velocity of the LP:

$$c_m(0) = \left(\frac{\beta}{2\pi^3}\right)^{\frac{1}{4}} \exp[-\beta(k_z^m - k_c)^2] \tag{21}$$

where $\beta = 10^{-12}$ m^2 is a coefficient characterising the shape of the wavepacket and $k_{z,m}$ is the in-plane momentum of polariton $|\psi_m\rangle$, given by:

$$\langle k_z^m \rangle = \frac{\sum_p^{n_{\text{mode}}} |\alpha_p^m|^2 k_{z,p}}{\sum_p^{n_{\text{mode}}} |\alpha_p^m|^2} \tag{22}$$

with $k_{z,p} = 2\pi p/L_z$ the discrete wavevector in a periodic 1D cavity of length L_z.

2.7.2 Off-resonant Excitation

In experiments off-resonant excitation is often realized by pumping a higher-energy electronic excited state of a single molecule. According to Kasha's rule, this molecule then rapidly relaxes into the lowest energy electronic excited state (S_1), which is resonant with the cavity. To avoid the computationally highly demanding description of this ultra-fast initial relaxation process, we started our simulations in the S_1 electronic state of one of the molecules. Because we performed our simulations in the adiabatic representation, in which the polaritonic wave functions are delocalized over all molecules, we took a linear combination of adiabatic states that localizes the excitation onto that molecule as initial conditions. To find the expansion coefficients for such localisation, we used the completeness relation to transform the adiabatic eigenstates of the Tavis-Cummings Hamiltonian ($|\psi^\alpha\rangle$, Equation 14) back into the basis of molecular excitations and cavity mode Fock

states (*i.e.*, $|\phi_j\rangle = \hat{\sigma}_j^+|\phi_0\rangle$ for $j \leq N$ and $|\phi_j\rangle = \hat{a}_j^\dagger|\phi_0\rangle$ for $i > N$):

$$\begin{aligned} \langle\psi^\alpha|\hat{H}^{\mathrm{TC}}|\psi^\beta\rangle &= \textstyle\sum_i \sum_j \langle\psi^\alpha|\phi_i\rangle\langle\phi_i|\hat{H}^{\mathrm{TC}}|\phi_j\rangle\langle\phi_j|\psi^\beta\rangle \\ &= \textstyle\sum_i \sum_j U_{\beta i}^\dagger\langle\phi_i|\hat{H}^{\mathrm{TC}}|\phi_j\rangle U_{j\alpha} \end{aligned} \tag{23}$$

where $\mathbf{H}^{\mathrm{TC}}$ is the matrix representation of the Tavis-Cummings Hamiltonian in Equation 1 with elements in Equations 8, 10 and 13, and $\mathbf{U}$ is the unitary matrix that diagonalizes $\mathbf{H}^{\mathrm{TC}}$ at the start of the simulation. Thus, the expansion coefficients for the linear combination of adiabatic states (Equation 15) that initially localizes the single-photon excitation onto molecule j are $c_\alpha(0) = U_{\alpha j}^\dagger$, *i.e.*:

$$|\Psi(0)\rangle^{(\text{loc. on } j)} = \sum_\alpha^{N+n_{\text{mode}}} c_\alpha(0)|\psi^\alpha\rangle = \sum_\alpha^{N+n_{\text{mode}}} U_{\alpha j}^\dagger|\psi^\alpha\rangle \tag{24}$$

3 Simulation Analysis

3.1 Contributions of Molecular and Cavity Mode Excitations to the Wavepacket

The total contribution of the molecular excitations ($|\Psi_{\text{exc}}|^2$) to the wavepacket (black curve in the fourth column of Figures 9, 10, 6 and 5 and panels **d** and **h** of Figures 2 and 4 of the main article), was obtained by summing the projections of the N excitonic basis states (*i.e.*, $|\phi_j\rangle = \hat{\sigma}_j^+|\phi_0\rangle$ for $1 \leq j \leq N$, Equation 7) onto $|\Psi(t)\rangle$ (Equation 15) at each time-step of the simulation:

$$\begin{aligned} |\Psi_{\text{exc}}(t)|^2 &= \textstyle\sum_{j=1}^{N} \left|\langle\phi_0|\hat{\sigma}_j|\Psi(t)\rangle\right|^2 \\ &= \textstyle\sum_{j=1}^{N} \left|\sum_{m}^{N+n_{\text{mode}}} c_m(t)\beta_j^m\right|^2 \end{aligned} \tag{25}$$

where $N + n_{\text{mode}}$ is the number of polaritonic states.

Likewise, the total contribution of the cavity mode excitations ($|\Psi_{\text{pho}}|^2$) to the wavepacket (red curve in the fourth column of Figures 9, 10, 6 and 5 and panels **d** and **h** of Figures 2 and 4 of the main article) was obtained by summing the projections of the n_{mode} cavity mode excitation basis states (*i.e.*, $|\phi_p\rangle = \hat{a}_p^\dagger|\phi_0\rangle$ for $0 \leq p < n_{\text{mode}}$) onto $|\Psi(t)\rangle$ (Equation 15) at each time step of the simulation:

$$\begin{aligned} |\Psi_{\text{pho}}(t)|^2 &= \textstyle\sum_{p=0}^{n_{\text{mode}}-1} \left|\langle\phi_0|\hat{a}_p|\Psi(t)\rangle\right|^2 \\ &= \textstyle\sum_{p=0}^{n_{\text{mode}}-1} \left|\sum_{m}^{N+n_{\text{mode}}} c_m(t)\alpha_p^m\right|^2 \end{aligned} \tag{26}$$

3.2 Monitoring Wavepacket Dynamics

To monitor the propagation of the wavepacket, we plotted the probability density of the total time-dependent wave function $|\Psi(t)|^2$ at the positions of the molecules, z_j, as a function of time (first column of Figures 9, 10, 6 and 5 and panels **a** and **e** of Figures 2 and 4 in the main text). We thus represent the density as a *discrete* distribution at grid points that correspond to the molecular positions, rather than a continuous distribution. In addition to the total probability density, $|\Psi(t)|^2$,

we also plotted the probability densities of the excitonic $|\Psi_{\text{exc}}(t)|^2$ and photonic $|\Psi_{\text{pho}}(t)|^2$ contributions separately (second and third columns of Figures 9, 10, 6 and 5 and panels **b**, **f** and **c**, **g**, respectively, of Figures 2 and 4 in the main text).

The amplitude of $|\Psi_{\text{exc}}(t)\rangle$ at position z_j in the 1D cavity (with $z_j = (j-1)L_z/N$ for $1 \leq j \leq N$) is obtained by projecting the excitonic basis state in which molecule j at position z_j is excited ($|\phi_j\rangle = \hat{\sigma}_j^+|\phi_0\rangle$) to the total wave function (Equation 15):

$$\begin{aligned} |\Psi_{\text{exc}}(z_j,t)\rangle &= \hat{\sigma}_j^+|\phi_0\rangle\langle\phi_0|\hat{\sigma}_j|\Psi(t)\rangle \\ &= \textstyle\sum_m^{N+n_{\text{mode}}} c_m(t)\beta_j^m\hat{\sigma}_j^+|\phi_0\rangle \end{aligned} \tag{27}$$

where the β_j^m are the expansion coefficients of the excitonic basis states in polaritonic state $|\psi^m\rangle$ (Equation 14) and $c_m(t)$ the time-dependent expansion coefficients of the total wavefunction $|\Psi(t)\rangle$ (Equation 15).

The cavity mode excitations are described as plane waves that are delocalized in real space. We therefore obtain the amplitude of the cavity modes in polaritonic eigenstate $|\psi^m\rangle$ at position z_j by Fourier transforming the projection of the cavity mode excitation basis states, in which cavity mode p is excited ($|\phi_p\rangle = \hat{a}_p^\dagger|\phi_0\rangle$):

$$\begin{aligned} |\psi_{\text{pho}}^m(z_j)\rangle &= \mathcal{FT}^{-1}\left[\textstyle\sum_p^{n_{\text{mode}}} \hat{a}_p^\dagger|\phi_0\rangle\langle\phi_0|\hat{a}_p|\psi^m\rangle\right] \\ &= \tfrac{1}{\sqrt{N}}\textstyle\sum_p^{n_{\text{mode}}} \alpha_p^m e^{i2\pi z_j p}\hat{a}_p^\dagger|\phi_0\rangle \end{aligned} \tag{28}$$

where the α_p^m are the expansion coefficients of the cavity mode excitations in polaritonic state $|\psi^m\rangle$ (Equation 14) and we normalize by $1/\sqrt{N}$ rather than $1/\sqrt{L_z}$, as we represent the density on the grid of molecular positions. The total contribution of the cavity mode excitations to the wavepacket

at position z_j at time t is then obtained as the weighted sum over the Fourier transforms:

$$|\Psi_{\text{pho}}(z_j,t)\rangle = \sum_m^{N+n_{\text{mode}}} c_m(t) \times \mathcal{FT}^{-1}\left[\sum_p^{n_{\text{mode}}} \hat{a}_p^\dagger|\phi_0\rangle\langle\phi_0|\hat{a}_p|\psi^m\rangle\right]$$
$$= \sum_m^{N+n_{\text{mode}}} c_m(t)\frac{1}{\sqrt{N}}\sum_p^{n_{\text{mode}}} \alpha_p^m e^{i2\pi z_j p}\hat{a}_p^\dagger|\phi_0\rangle \quad (29)$$

with $c_m(t)$ the time-dependent expansion coefficients of the adiabatic polaritonic states $|\psi^m\rangle$ in the total wavefunction $|\Psi(t)\rangle$ (Equation 15).

3.3 Average Velocity

To determine the average velocity, v_{av}, of the total wave packet, we first computed the expectation value of the position as a function of time:

$$\langle z(t)\rangle = \frac{\langle\Psi(t)|\hat{z}|\Psi(t)\rangle}{\langle\Psi(t)|\Psi(t)\rangle}. \quad (30)$$

Then, v_{av} was obtained from the slope of a linear fit to $\langle z(t)\rangle$.

3.4 Photo-Absorption Spectra

Following Lidzey and coworkers,[46] we define the "visibility", I^m, of polaritonic state ψ^m as the total photonic contribution to that state (*i.e.*, $I^m \propto \sum_p^{n_{\text{max}}} |\alpha_p^m|^2$). Thus, the angle-resolved, or wave vector-dependent, one-photon absorption spectra of the Rhodamine cavity systems were computed from the QM/MM trajectory of the uncoupled Rhodamine as follows: For each frame of this trajectory, the polaritonic states were computed and the energy gaps of these states with respect to the overall ground state (*i.e.*, E^0, with all molecules in S_0, no photon in the cavity, Equation 7), were extracted for all cavity modes, p, and summed up into a superposition of Gaussian functions,

weighted by $|\alpha_p^m|^2$:

$$I^{\text{abs}}(E, k_z) \propto \sum_t^s \left[\sum_m^{N+n_{\text{max}}+1} |\alpha_{p,t}^m|^2 \exp[-\frac{(E - \Delta E_t^m)^2}{2\sigma^2}] \right] \tag{31}$$

Here, $I^{\text{abs}}(E, k_z)$ is the absorption intensity as a function of excitation energy E and in-plane momentum $k_z = 2\pi p/L_z$, s the number of trajectory frames included in the analysis, ΔE_t^m the excitation energy of polaritonic state m in frame t (*i.e.*, $\Delta E_t^m = E_t^m - E_t^0$) and $\alpha_{p,t}^m$ the expansion coefficient of cavity mode p in polaritonic state m in that frame (equation 14). A width of $\sigma = 0.05$ eV was chosen for the convolution.

3.5 Photo-Luminescence Spectra

As in Berghuis *et al.*,[37] we analysed the photo-luminescence of the propagating wavepacket after non-resonant excitation through a (virtual) mask placed at various distances from the molecule that was excited initially. This analysis thus mimics the Fourier Transform microscopy technique that is used experimentally to determine how different polaritonic states contribute to the propagation.[37] In such imaging experiments, the photoluminescence from a specific area of the sample is collected through a pinhole and converted into momentum space. In the analysis of our simulations we modeled this pinhole, or mask, as a rectangular function of width $\Delta z = 10$ μm centered at $z = 10$ and 20 μm. At each time-step t, the cavity photoluminescence at the position of the pinhole was collected as follows:

First, the excitonic (Equation 27) and cavity mode contributions (Equation 29) to the wave

function (Equation 15) were convoluted with the rectangular mask function:

$$|\Psi_{\text{exc}}^{\text{hole}}(z,t)\rangle = \sum_{z_j \geq z-\Delta z/2}^{z_j \leq z+\Delta z/2} |\Psi_{\text{exc}}(z_j,t)\rangle \tag{32}$$

$$\tag{33}$$

$$|\Psi_{\text{pho}}^{\text{hole}}(z,t)\rangle = \sum_{z_j \geq z-\Delta z/2}^{z_j \leq z+\Delta z/2} |\Psi_{\text{pho}}(z_j,t)\rangle \tag{34}$$

Second, $|\Psi_{\text{pho}}^{\text{hole}}(z,t)\rangle$ was Fourier transformed from real space into k_z space:

$$|\Psi_{\text{pho}}^{\text{hole}}(k_z,t)\rangle = \mathcal{FT}\left[\Psi_{\text{pho}}^{\text{hole}}(z,t)\right] \tag{35}$$

such that the total wave function under the mask can be written as $|\Psi^{\text{hole}}(t)\rangle = |\Psi_{\text{exc}}^{\text{hole}}(z,t)\rangle \otimes |\Psi_{\text{pho}}^{\text{hole}}(k_z,t)\rangle$. Since the polaritonic states $\{|\psi^m\rangle\}$ form a complete basis (Equation 15), the part of the wavepacket under the mask at time t can be expanded as a linear combination of these eigenstates:

$$|\Psi^{\text{hole}}(t)\rangle = \sum_{m}^{N+n_{\text{mode}}} d_m(t)|\psi^m\rangle \tag{36}$$

To obtain the $N + n_{\text{mode}}$ expansion coefficients, $d_m(t)$, we computed the overlap between the polaritonic eigenstates and the fraction of the wavepacket visible through the pinhhole:

$$d_m(t) = \langle\psi^m|\Psi^{\text{hole}}(z,t)\rangle \tag{37}$$

Following Lidzey and co-workers,[46] we approximated the photoluminescence detected through the pinhole at time t as the "visibility" of the polaritonic states weighted by their expansion coefficients under the mask, $d_m(t)$:[5]

$$I_{\text{hole}}(E,p,z,t) \propto \sum_{m}^{N+n_{\text{mode}}} |d_m(t)|^2|\alpha_p^m|^2 \exp\left[-\frac{(E-\Delta E^m)^2}{2\sigma^2}\right] \tag{38}$$

with $I_{\text{hole}}(E, p, z, t)$ the photoluminescence intensity at time t as a function of excitation energy E, in-plane momentum p ($k_{z,p} = 2\pi p/L_z$) and position of the mask z; ΔE^m the excitation energy of polaritonic state $|\psi^m\rangle$ at that time frame ($\Delta E^m = E^m - E^0$, with E^0 the energy of the ground state, $|\phi_0\rangle$, Equation 7); and $\sigma = 0.05$ eV the convolution width. The visibility through the pinhole, $I_{\text{hole}}(E, p, z)$, was accumulated over 100 fs:

$$I_{\text{hole}}(E, p, z) = \sum_t^s I_{\text{hole}}(E, p, z, t) \tag{39}$$

where s is the number of time steps (1000 used for the pinhole analysis of simulations under off-resonant excitation conditions with no photon losses in this work).

4 Supplementary Results and Discussion

4.1 Polariton Transport for Different Numbers of Molecules N

Because the number of molecules we can include in our simulations is necessarily much smaller than in experiments, we investigated how that number affects energy transport by performing simulations with 160, 256, 368, 512, 768 and 1024 molecules. To keep the Rabi splitting ($\sim$ 325 meV) constant, and hence polariton dispersion the same, we scaled the cavity mode volume by the number of molecules N (see Section 2 for details).

4.1.1 On-resonant Excitation

In Supplementary Figure 5 we show the time-evolution of the probability density of the polaritonic wave function, $|\Psi(t)|^2$, after resonant excitation of a Gaussian wavepacket of LP states with a broad-band laser pulse in a perfect lossless cavity ($\gamma = 0$ ps^{-1}, Equation 19) containing 256, 512 and 1024 Rhodamine molecules. In Supplementary Figure 6 we show the time evolution of the probability density of $|\Psi(t)|^2$ after resonant excitation in a lossy cavity ($\gamma = 66.7$ ps^{-1} for all cavity modes) containing 256, 512 and 1024 Rhodamine molecules. In Figures 7 and 8 we plot the expectation value of the position, $\langle z \rangle$, and mean square displacement (MSD) of the total time-dependent wavefunction, $|\Psi(t)\rangle$, for $N = 256$ and 512 molecules under on-resonant excitation conditions in the lossless and lossy cavities, respectively. Plots of $\langle z \rangle$ and MSD for 1024 molecules are discussed in the main text (Figure 3).

The underlying polariton-mediated transport mechanism is the same for all N: Initially the propagation is ballistic and the wavepacket moves with the central group velocity until the contribution of the molecular excitons to the total wavefunction (Equation 15) exceeds the contributions of the cavity mode excitations. Then, the propagation continues as a diffusion process, as indicated by the change in the slope of $\langle z \rangle$ and the change from a quadratic to a linear time-dependence in the Mean Square Displacement (Figures 7 and 8).

When photon loss is included (Supplementary Figure 6), population builds up in the ground

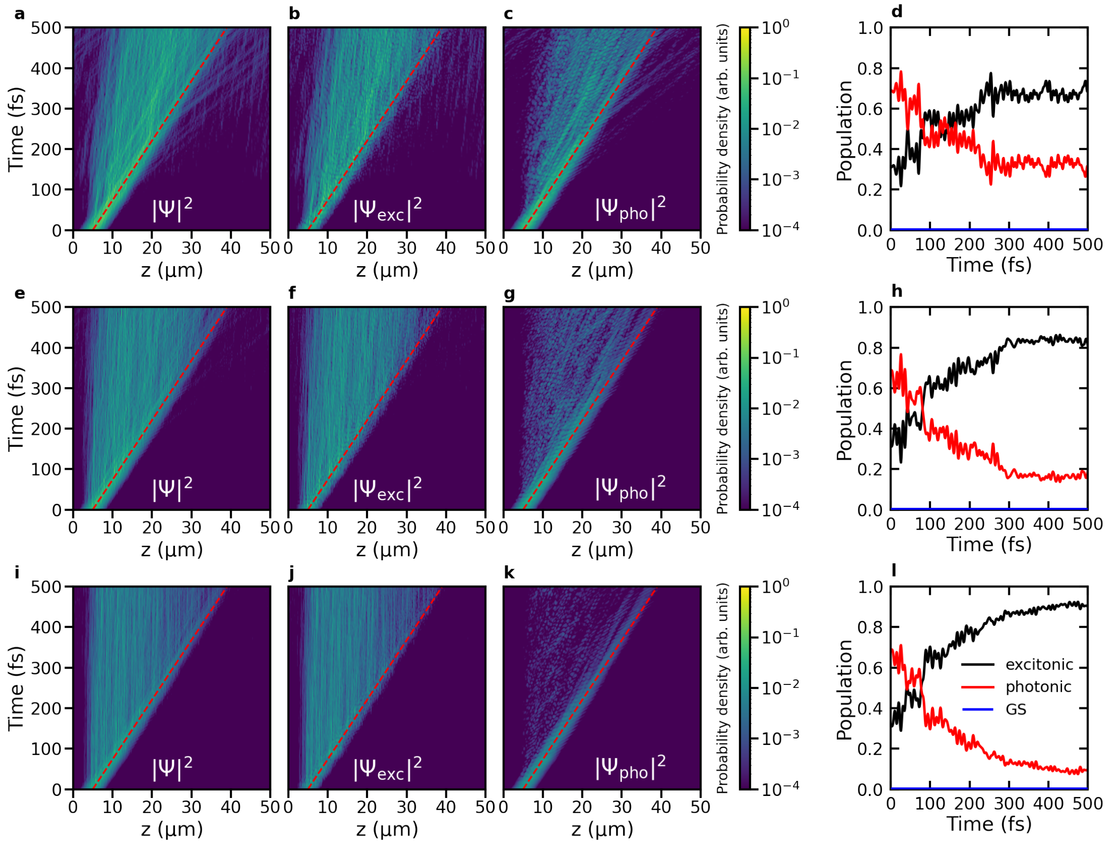

Supplementary Figure 5: Polariton propagation in ensembles of 256 (**a**-**d**), 512 (**e**-**h**) and 1024 molecules (**i**-**l**) strongly coupled to a lossless cavity (*i.e.*, $\gamma_{\text{cav}} = 0$ ps^{-1}), after on-resonant excitation of a wavepacket of lower polariton (LP) states centered at the k_z-vector corresponding to the maximum LP group velocity. Panel **a**, **b**, **c**: total probability density, $|\Psi(z,t)|^2$, probability density of the molecular excitons, $|\Psi_{\text{exc}}(z,t)|^2$, and of the cavity mode excitations, $|\Psi_{\text{pho}}(z,t)|^2$, respectively, as a function of distance (z, horizonal axis) and time (vertical axis) in the ensemble of 256 molecules. The red dashed line indicates propagation at the maximum group velocity of the LP (68 μmps^{-1}). Panel **d**: Contributions of the molecular excitons (black) and cavity mode excitations (red) to $|\Psi(z,t)|^2$ as a function of time in the ensemble of 256 molecules. Without cavity decay, the population of the ground state (GS, blue) remains zero. Panel **e**, **f**, **g**: total probability density, $|\Psi(z,t)|^2$, probability density of the molecular excitons, $|\Psi_{\text{exc}}(z,t)|^2$, and of the cavity mode excitations, $|\Psi_{\text{pho}}(z,t)|^2$, respectively, as a function of distance and time in the ensemble of 512 molecules. Panel **h**: Contributions of the molecular excitons (black) and cavity mode excitations (red) to $|\Psi(z,t)|^2$ as a function of time in the ensemble of 512 molecules. Panel **i**, **j**, **k**: total probability density, $|\Psi(z,t)|^2$, probability density of the molecular excitons, $|\Psi_{\text{exc}}(z,t)|^2$, and of the cavity mode excitations, $|\Psi_{\text{pho}}(z,t)|^2$, respectively, as a function of distance and time in the ensemble of 1024 molecules. Panel **l**: Contributions of the molecular excitons (black) and cavity mode excitations (red) to $|\Psi(z,t)|^2$ as a function of time in the ensemble of 1024 molecules.

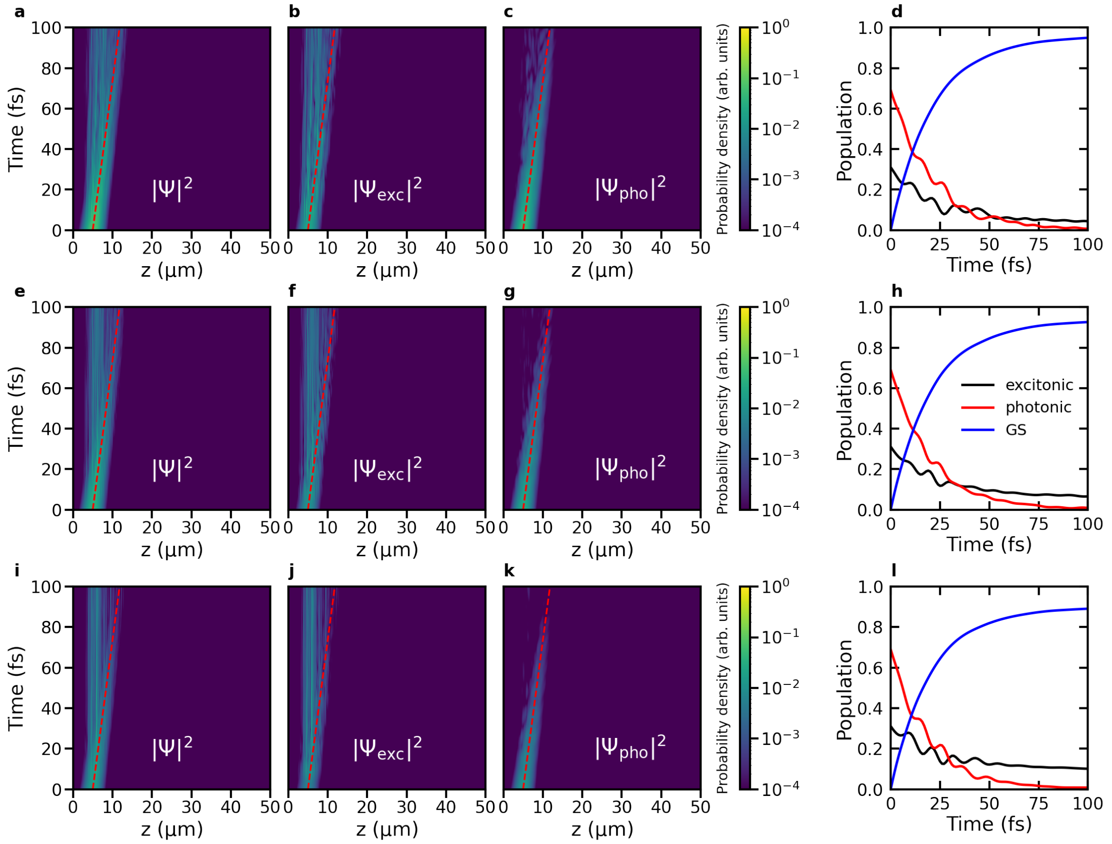

Supplementary Figure 6: Polariton propagation in an ensemble of 256 (**a-d**), 512 (**e-h**) and 1024 molecules (**i-l**) strongly coupled to a lossy cavity (*i.e.*, $\gamma_{\mathrm{cav}} = 66.7$ ps^{-1}), after on-resonant excitation of a wavepacket of lower polariton (LP) states centered at the k_z-vector corresponding to the maximum LP group velocity. Panel **a**, **b**, **c**: total probability density, $|\Psi(z,t)|^2$, probability density of the molecular excitons, $|\Psi_{\mathrm{exc}}(z,t)|^2$, and of the cavity mode excitations, $|\Psi_{\mathrm{pho}}(z,t)|^2$, respectively, as a function of distance (z, horizontal axis) and time (vertical axis). The red dashed line indicates propagation at the maximum group velocity of the LP (68 μmps^{-1}). Panel **d**: Contributions of molecular excitons (black) and cavity mode excitations (red) to $|\Psi(z,t)|^2$ and population of the ground state (GS, blue) as a function of time. Panel **e**, **f**, **g**: total probability density, $|\Psi(z,t)|^2$, probability density of the molecular excitons, $|\Psi_{\mathrm{exc}}(z,t)|^2$, and of the cavity mode excitations, $|\Psi_{\mathrm{pho}}(z,t)|^2$, respectively, as a function of distance and time in the ensemble of 512 molecules. Panel **h**: Contributions of the molecular excitons (black) and cavity mode excitations (red) to $|\Psi(z,t)|^2$ as a function of time in the ensemble of 512 molecules. Panel **i**, **j**, **k**: total probability density, $|\Psi(z,t)|^2$, probability density of the molecular excitons, $|\Psi_{\mathrm{exc}}(z,t)|^2$, and of the cavity mode excitations, $|\Psi_{\mathrm{pho}}(z,t)|^2$, respectively, as a function of distance and time in the ensemble of 1024 molecules. Panel **l**: Contributions of the molecular excitons (black) and cavity mode excitations (red) to $|\Psi(z,t)|^2$ as a function of time in the ensemble of 1024 molecules.

state (blue curves) at the expense of states with cavity mode excitations (red). Because the number of dark states increases with N, and the dark states "protect" against decay, the total decay decreases

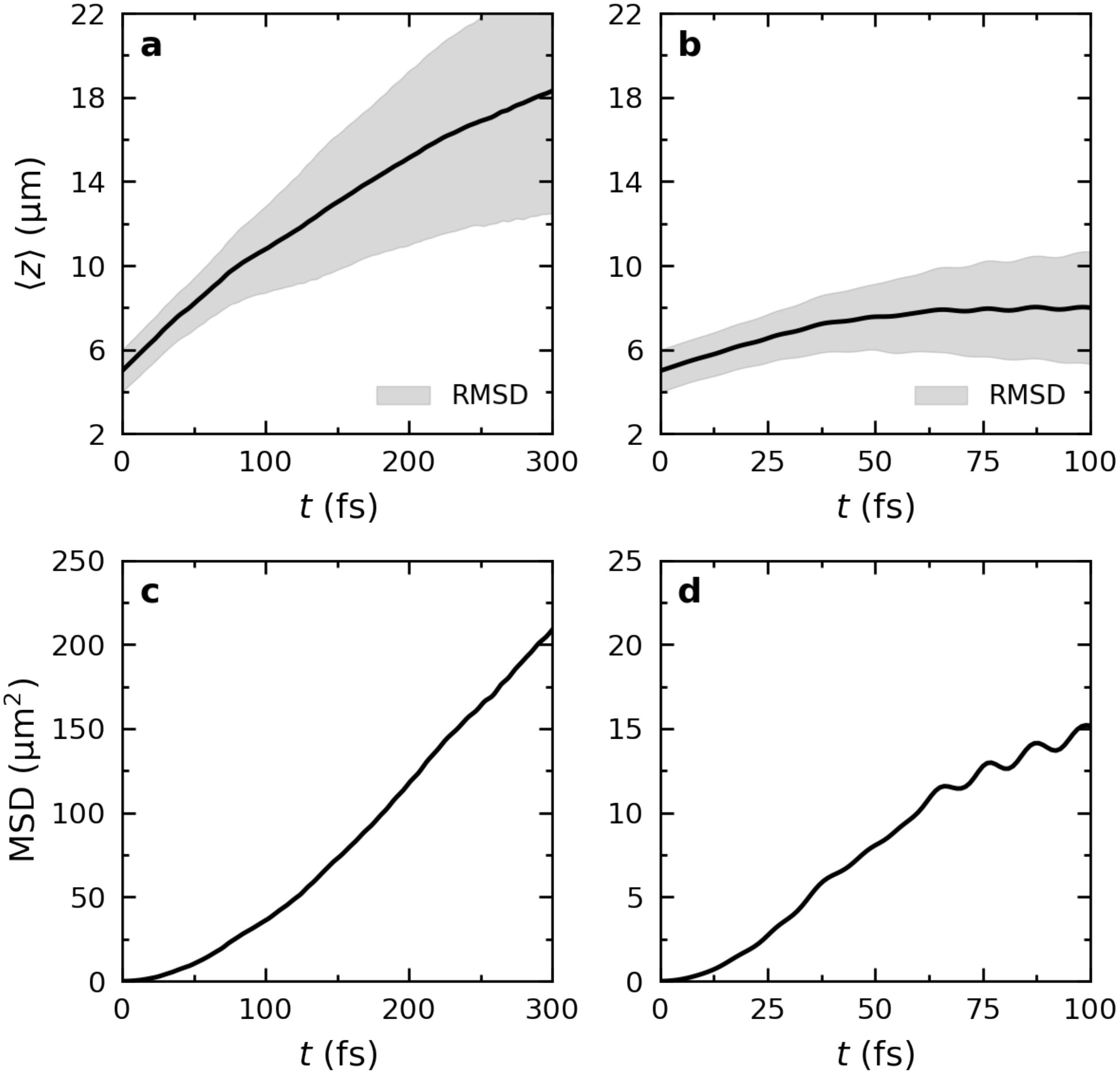

Supplementary Figure 7: Expectation value of the position ($\langle z\rangle = \frac{\langle\Psi(z,t)|\hat{z}|\Psi(z,t)\rangle}{\langle\Psi(z,t)|\Psi(z,t)\rangle}$) (top panels) and mean square displacement (MSD, *i.e.*, $\langle(z(t) - \langle z(0)\rangle)^2\rangle$) (bottom panels) of the total time-dependent wavefunction $|\Psi(z,t)\rangle$ of 256 Rhodamine chromophores strongly coupled to an ideal cavity (*i.e.*, $\gamma_{\rm cav} = 0$ ps^{-1}, panels **a** and **c**) and a lossy cavity with $\gamma_{\rm cav} = 66.7$ ps^{-1} (panels **b** and **d**), under on-resonant excitation conditions. In the top panels, the black lines represent $\langle z\rangle$ while the shaded area indicates the root mean squared deviation (RMSD, *i.e.*, $\sqrt{\langle(z(t) - \langle z(t)\rangle)^2\rangle}$).

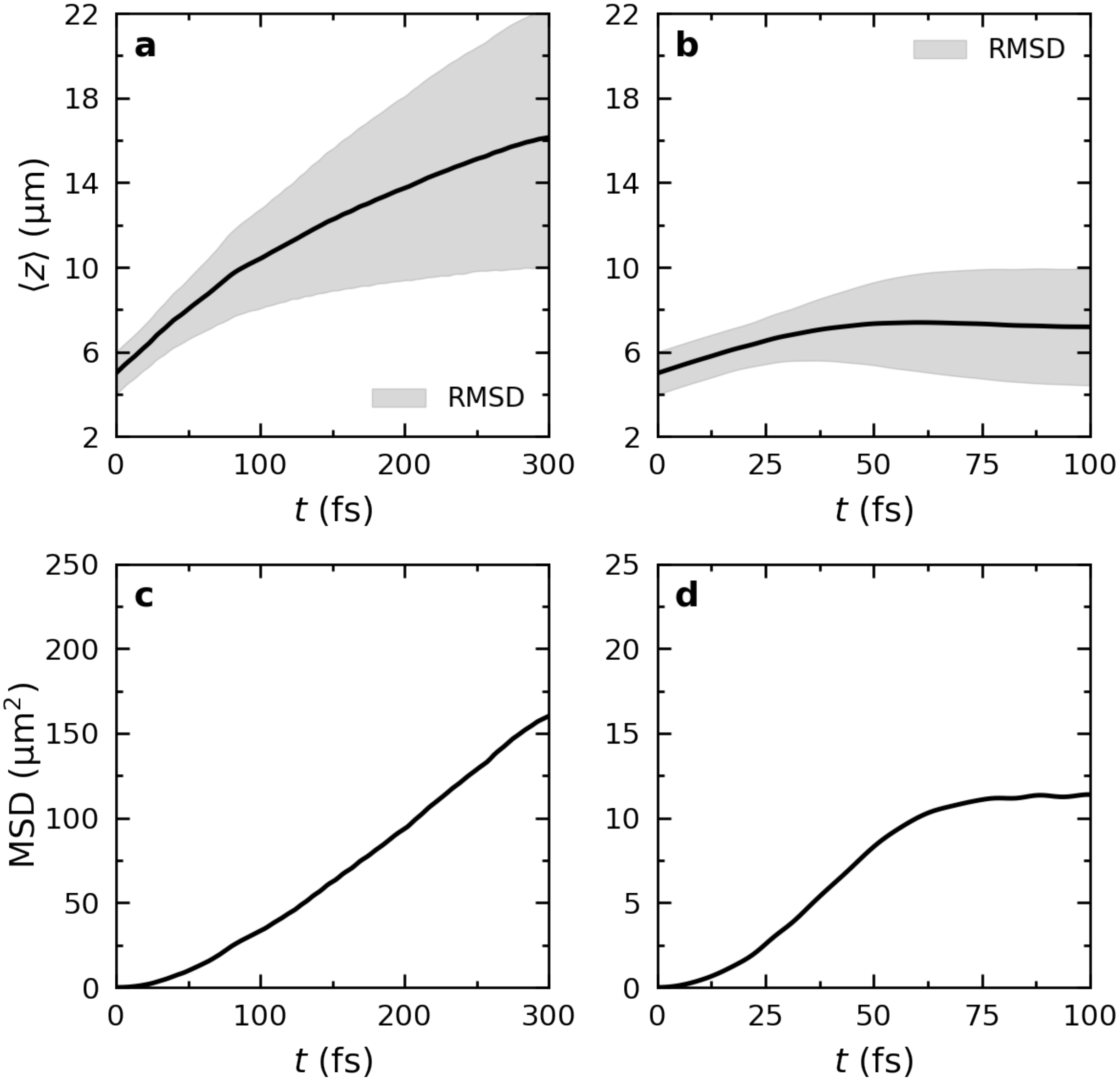

Supplementary Figure 8: Expectation value of the position ($\langle z\rangle=\frac{\langle\Psi(z,t)|\hat{z}|\Psi(z,t)\rangle}{\langle\Psi(z,t)|\Psi(z,t)\rangle}$) (top panels) and mean square displacement (MSD, *i.e.*, $\langle(z(t)-\langle z(0)\rangle)^2\rangle$) (bottom panels) of the total time-dependent wavefunction $|\Psi(z,t)\rangle$ of 512 Rhodamine chromophores strongly coupled to an ideal cavity (*i.e.*, $\gamma_{\rm cav}=0$ ps^{-1}, panels **a** and **c**) and a lossy cavity with $\gamma_{\rm cav}=66.7$ ps^{-1} (panels **b** and **d**), under on-resonant excitation conditions. In the top panels, the black lines represent $\langle z\rangle$ while the shaded area indicates the root mean squared deviation (RMSD, *i.e.*, $\sqrt{\langle(z(t)-\langle z(t)\rangle)^2\rangle}$).

with N, albeit that for the ensemble sizes simulated here, the effect is rather small.

4.1.2 Off-resonant Excitation

In Supplementary Figure 9 we show the propagation of the wavepacket after off-resonant excitation in a loss-less cavity ($\gamma = 0$ ps^{-1}, Equation 19) with 160, 256, 512 and 1024 Rhodamine molecules. In Supplementary Figure 10 we show the propagation of the wavepacket after off-resonant excitation in a lossy cavity ($\gamma = 66.7$ ps^{-1} for all cavity modes) with 256, 512 and 1024 Rhodamine molecules.

Because the initial wavefunction that localizes the excitation onto a single molecule at the start of the simulation, is a superposition of all states, including the highest-energy, highest-momentum UP states, the fastest propagation in the smaller ensembles (*i.e.*, $N < 512$) is dominated by wavepackets of these states. Since UP states posses much higher group velocities compared to LP states, these wavepackets cross the periodic boundary around 200 fs and start self-interfering in these ensembles. We therefore only plot $\langle z \rangle$ and MSD until such crossings occur in Figures 11, 12 and 13.

In the smallest ensemble, with 160 molecules coupled to the 160 modes of the 1D Fabry-Pérot cavity, there are no dark states and all states are hybrids of molecular and cavity mode excitations. Therefore, in contrast to the larger ensembles with dark states, all states have group velocity due to their cavity mode excitations, and all of the population propagates ballistically, as evidenced by the quadratic dependence of the mean-square displacement (MSD) on time (Supplementary Figure 11). Because in the largest ensembles with many dark states, the propagation appears diffusive, the 160 molecule simulation *without* dark states provides additional support for the conclusion that transient trapping of the population in stationary dark states is the origin for such diffusion.

Comparing the relative contributions to the wavepacket of the molecular excitons (black) and cavity mode excitations (red) in the population plots for 160, 256, 512 and 1024 Rhodamine molecules in the right column of Supplementary Figure 9 suggests that as the number of molecules, and hence the number of dark states, increases, the total population that resides in bright light-matter states decreases. As explained in Tichauer *et al.*[47], such decrease in bright state population is due to $1/N$ scaling of the rate at which population transfers between dark and bright states.[47] Because the number of dark states is proportional to N, while (for large N) the number of bright states is constant, this dependency affects the ratio between the population in the dark and bright states, with

the latter rapidly decreasing with increasing N.

With a larger fraction of the population residing in the stationary dark states, the wavepacket propagates more slowly, as evidenced by comparing the expectation value of $\langle z \rangle$ in Supplementary Figure 11 for 160 molecules (without dark states), Supplementary Figure 12 for 256 molecules, Supplementary Figure 13 for 512 molecules and Supplementary Figure 5 (main text) for 1024 molecules. Average propagation velocities (υ_{av}) obtained by fitting a linear function to $\langle z \rangle$, are plotted as a function of the number of molecules in Supplementary Figure 14. In this plot we also include the average propagation velocity obtained for additional simulations with 384 and 768 Rhodamines strongly coupled to the cavity with the same Rabi splitting. Because the overall propagation velocity is determined by the population of the bright states, and that population is inversely proportional to N due to the inverse scaling of the non-adiabatic population transfer rate,[47] also the velocity is inversely proportional to N. Indeed, fitting the average velocities to $\upsilon_{\text{av}}(N) = a/\left(N + b\right)$ yields a satisfactory description of the data (dashed line in Supplementary Figure 14).

Because of the $1/N$ scaling, the value of υ_{av} approaches the "thermodynamic limit" already around 1000 molecules. We therefore consider the results of the simulations with 1024 Rhodamines sufficiently representative for experiment and for providing qualitative insights into polariton propagation. Indeed, the υ_{av} of 9.6 μmps^{-1} in the cavity containing 1024 molecules is about an order of magnitude below the maximum group velocity of the LP (68 μmps^{-1}) in line with experiments on organic microcavities,[42] and cavity-free polaritons.[48]

Furthermore, with a larger population in bright states, the motion of the wavepacket in the smaller ensembles is also more ballistic, as shown in the Mean-Square Displacement (MSD) plots in Figures 11, 12, 13, and Figure 3 of the main text for 160, 256, 512, and 1024 molecules, respectively. For the smaller ensembles the MSD increases more quadratically with time, in contrast to the largest ensemble with 1024 Rhodamine molecules, for which a more linear dependency is observed (Supplementary Figure 3**c**, main text). Because a linear dependency of the MSD on time is associated with diffusion, these trends further confirm that stationary dark states are responsible for diffusive propagation.

When cavity losses are added, population transfer into the ground state competes with polariton propagation (Supplementary Figure 10). Because only states with a significant contribution from cavity mode excitations can emit, the overall population decays faster for the smaller ensembles, suggesting that dark states can "protect" the excitation from decay under off-resonance excitation conditions and hence increase the lifetime of the excitation.[21]

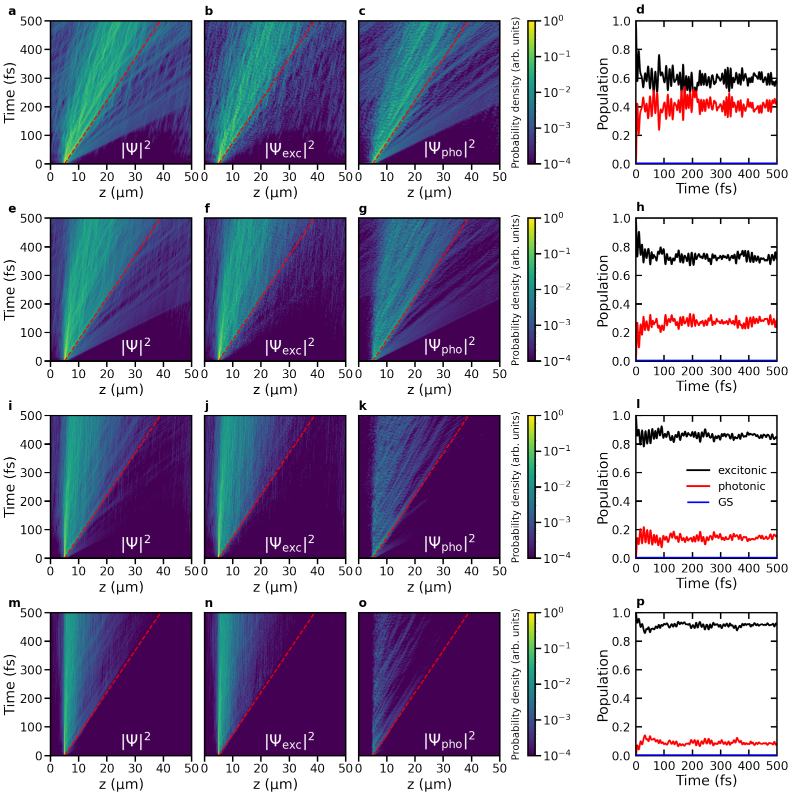

Supplementary Figure 9: Polariton propagation after off-resonant excitation into the S_1 state of a single molecule located at 5 μm in an ensemble of 160 (**a**-**d**), 256 (**e**-**h**), 512 (**i**-**l**) and 1024 (**m**-**p**) molecules strongly coupled to 160 confined light modes of a 1D Fabry-Pérot optical cavity with perfect mirrors (*i.e.*, $\gamma_{\text{cav}} = 0\ \text{ps}^{-1}$). Panel **a**, **b**, **c**: total probability density, $|\Psi(z,t)|^2$, probability density of the molecular excitons, $|\Psi_{\text{exc}}(z,t)|^2$, and of the cavity mode excitations, $|\Psi_{\text{pho}}(z,t)|^2$, respectively, as a function of distance (z, horizontal axis) and time (vertical axis) in the ensemble of 160 molecules. The red dashed line indicates propagation at the maximum group velocity of the lower polaritons (68 μmps^{-1}). Panel **d**: Contributions of the molecular excitons (black) and cavity mode excitations (red) to $|\Psi(z,t)|^2$ as a function of time in the ensemble of 160 molecules. Without cavity decay, the population of the ground state (GS, blue) remains zero. Panel **e**, **f**, **g**: total probability density, $|\Psi(z,t)|^2$, probability density of the molecular excitons, $|\Psi_{\text{exc}}(z,t)|^2$, and of the cavity mode excitations, $|\Psi_{\text{pho}}(z,t)|^2$, respectively, as a function of distance and time in the ensemble of 256 molecules. Panel **h**: Contributions of the molecular excitons (black) and cavity mode excitations (red) to $|\Psi(z,t)|^2$ as a function of time in the ensemble of 256 molecules. Panel **i**, **j**, **k**: total probability density, $|\Psi(z,t)|^2$, probability density of the molecular excitons, $|\Psi_{\text{exc}}(z,t)|^2$, and of the cavity mode excitations, $|\Psi_{\text{pho}}(z,t)|^2$, respectively, as a function of distance and time in the ensemble of 512 molecules. Panel **l**: Contributions of the molecular excitons (black) and cavity mode excitations (red) to $|\Psi(z,t)|^2$ as a function of time in the ensemble of 512 molecules. Panel **m**, **n**, **o**: total probability density, $|\Psi(z,t)|^2$, probability density of the molecular excitons, $|\Psi_{\text{exc}}(z,t)|^2$, and of the cavity mode excitations, $|\Psi_{\text{pho}}(z,t)|^2$, respectively, as a function of distance and time in the ensemble of 1024 molecules. Panel **p**: Contributions of the molecular excitons (black) and cavity mode excitations (red) to $|\Psi(z,t)|^2$ as a function of time in the ensemble of 1024 molecules.

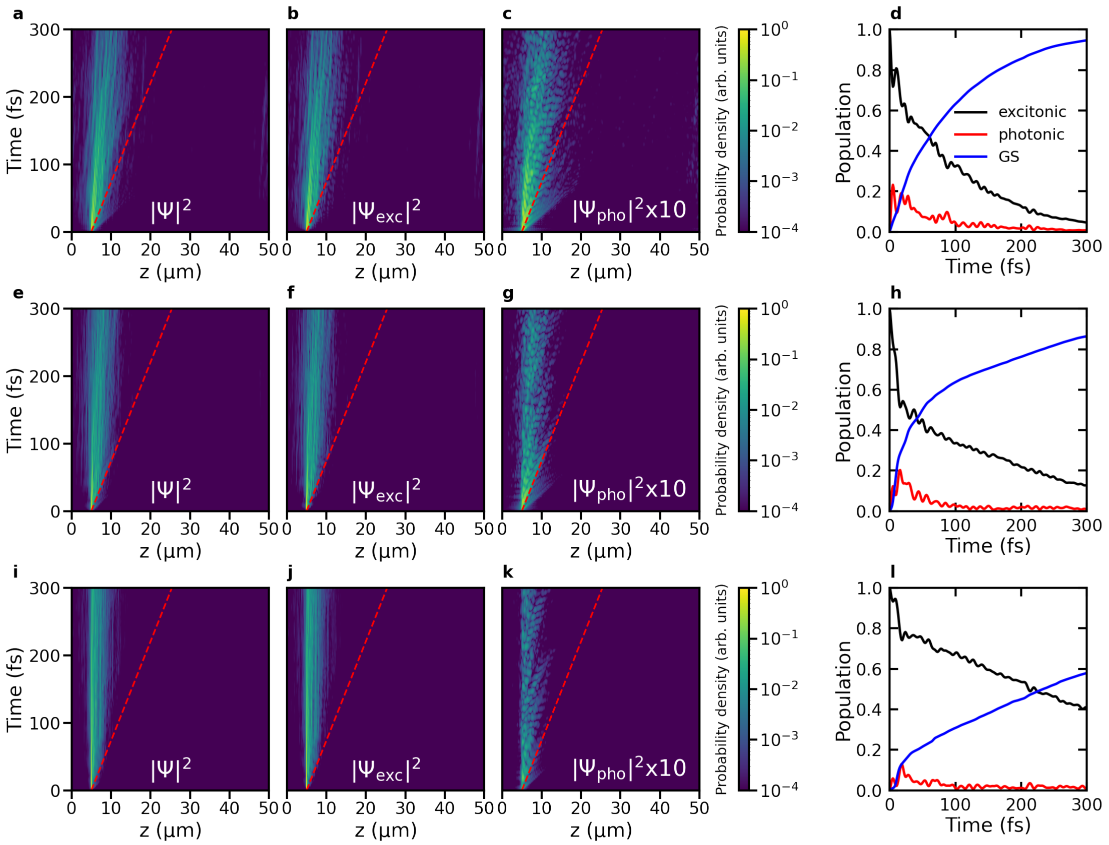

Supplementary Figure 10: Polariton propagation after off-resonant excitation into the S_1 state of a single molecule located at 5 µm in an ensemble of 256 (**a**-**d**), 512 (**e**-**h**) and 1024 molecules (**i**-**l**)) strongly coupled to 160 confined light modes of a 1D Fabry-Pérot optical cavity with lossy mirrors (*i.e.*, $\gamma_{\text{cav}} = 66.7$ ps^{-1}). Panel **a**, **b**, **c**: total probability density, $|\Psi(z,t)|^2$, probability density of the molecular excitons, $|\Psi_{\text{exc}}(z,t)|^2$, and of the cavity mode excitations, $|\Psi_{\text{pho}}(z,t)|^2$, respectively, as a function of distance (z, horizontal axis) and time (vertical axis) in the ensemble of 256 molecules. The red dashed line indicates propagation at the maximum group velocity of the lower polaritonis (68 μmps^{-1}). Panel **d**: Contributions of molecular excitons (black) and cavity mode excitations (red) to $|\Psi(z,t)|^2$ and population of the ground state (GS, blue) as a function of time in the ensemble of 256 molecules. Panel **e**, **f**, **g**: total probability density, $|\Psi(z,t)|^2$, probability density of the molecular excitons, $|\Psi_{\text{exc}}(z,t)|^2$, and of the cavity mode excitations, $|\Psi_{\text{pho}}(z,t)|^2$, respectively, as a function of distance and time in the ensemble of 512 molecules. Panel **h**: Contributions of molecular excitons (black) and cavity mode excitations (red) to $|\Psi(z,t)|^2$ and population of the ground state (GS, blue) as a function of time in the ensemble of 512 molecules. Panel **i**, **j**, **k**: total probability density, $|\Psi(z,t)|^2$, probability density of the molecular excitons, $|\Psi_{\text{exc}}(z,t)|^2$, and of the cavity mode excitations, $|\Psi_{\text{pho}}(z,t)|^2$, respectively, as a function of distance and time in the ensemble of 1024 molecules. Panel **l**: Contributions of molecular excitons (black) and cavity mode excitations (red) to $|\Psi(z,t)|^2$ and population of the ground state (GS, blue) as a function of time in the ensemble of 1024 molecules.

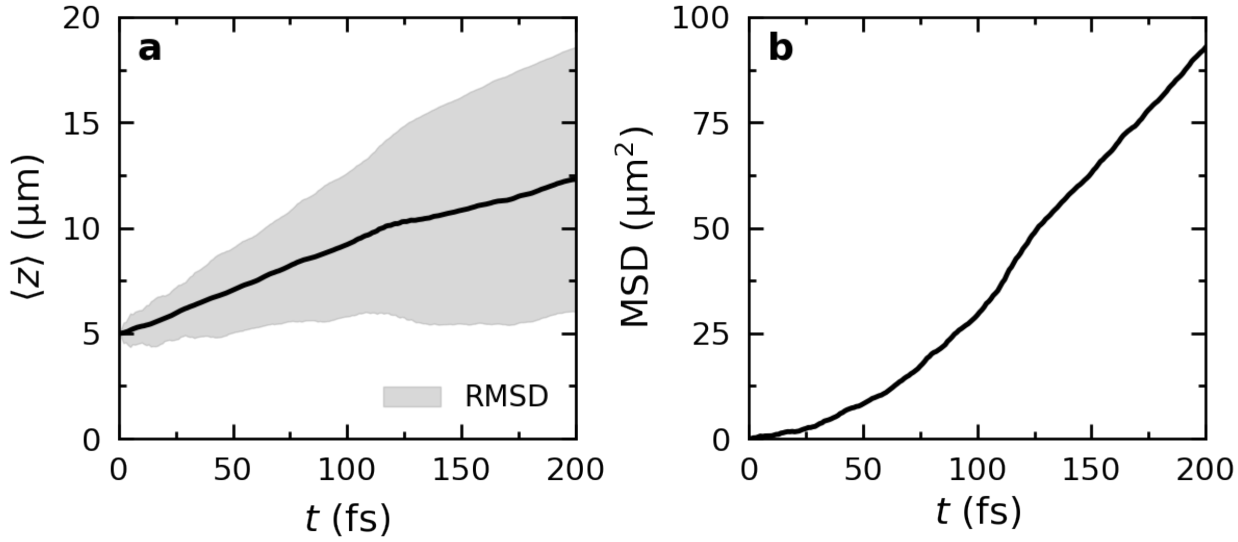

Supplementary Figure 11: Expectation value of the position ($\langle z\rangle$=$\langle\Psi(z,t)|\hat{z}|\Psi(z,t)\rangle/\langle\Psi(z,t)|\Psi(z,t)\rangle$, panel **a**) and mean squared displacement (MSD, *i.e.*, $\langle(z(t)-\langle z(0)\rangle)^2\rangle$, panel **b**) of the total time-dependent wavefunction $|\Psi(z,t)\rangle$ of 160 Rhodamine molecules strongly coupled to a perfect Fabry-Pérot cavity (*i.e.*, $\gamma_{\text{cav}} = 0$ ps^{-1}), after off-resonant excitation into the S_1 state of a single molecule located at 5 μm. In panel **a**, the black line represents $\langle z\rangle$ while the shaded area shows the root mean squared deviation (RMSD, *i.e.*, $\sqrt{\langle(z(t)-\langle z(t)\rangle)^2\rangle}$).

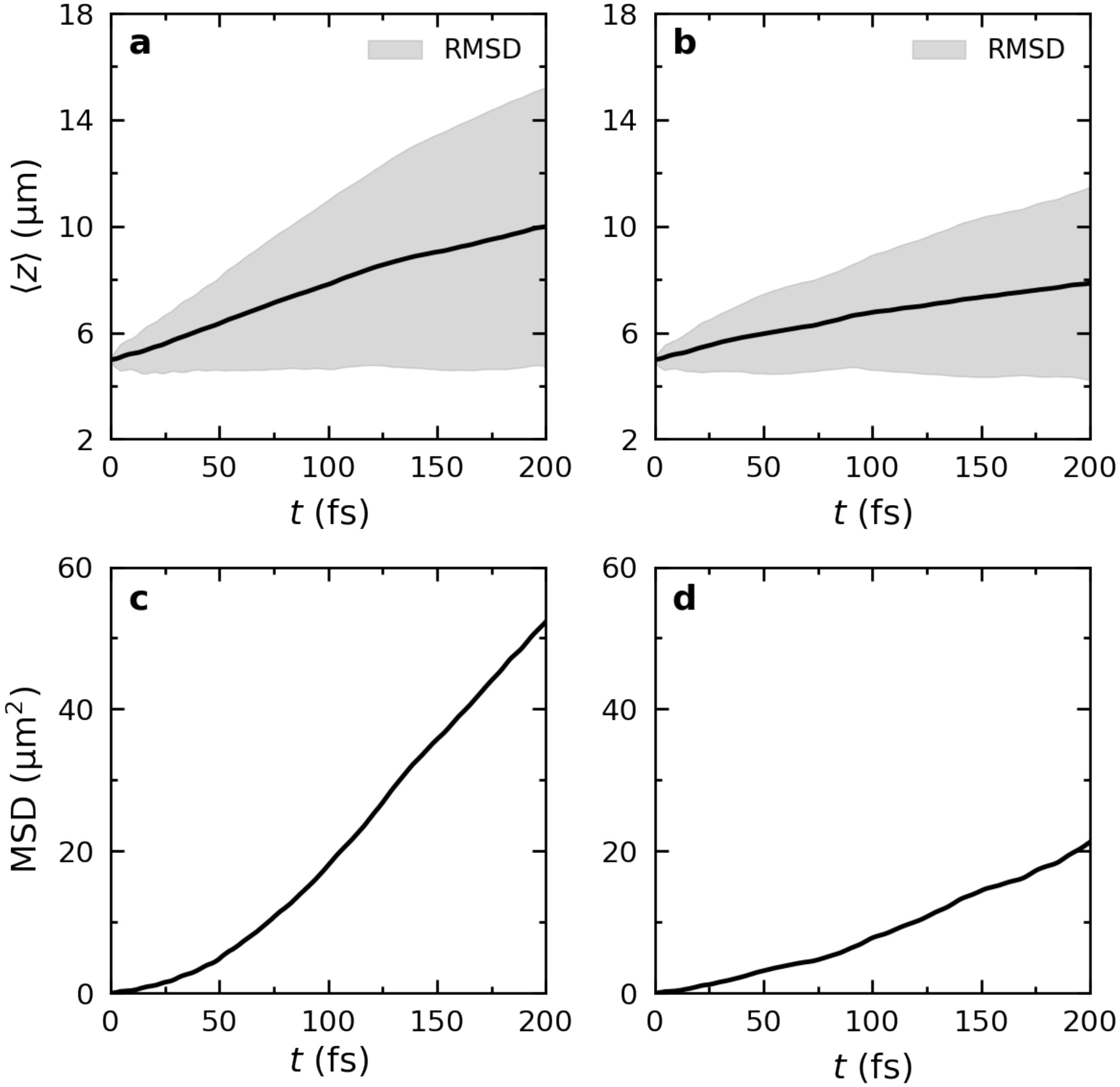

Supplementary Figure 12: Expectation value of the position ($\langle z\rangle=\frac{\langle\Psi(z,t)|\hat{z}|\Psi(z,t)\rangle}{\langle\Psi(z,t)|\Psi(z,t)\rangle}$, top panels) and mean squared displacement (MSD, *i.e.*, $\langle(z(t)-\langle z(0)\rangle)^2\rangle$, bottom panels) of the total time-dependent wavefunction $|\Psi(z,t)\rangle$ of 256 Rhodamine molecules strongly coupled to an ideal cavity *i.e.*, $\gamma_{\text{cav}}=0$ ps^{-1} (panels **a** and **c**) and a lossy cavity with $\gamma_{\text{cav}}=66.7$ ps^{-1} (panels **b** and **d**), after off-resonant excitation of a single molecule. In the top panels, the black lines represents $\langle z\rangle$ while the shaded area shows the root mean squared deviation (RMSD, *i.e.,* $\sqrt{\langle(z(t)-\langle z(t)\rangle)^2\rangle}$).

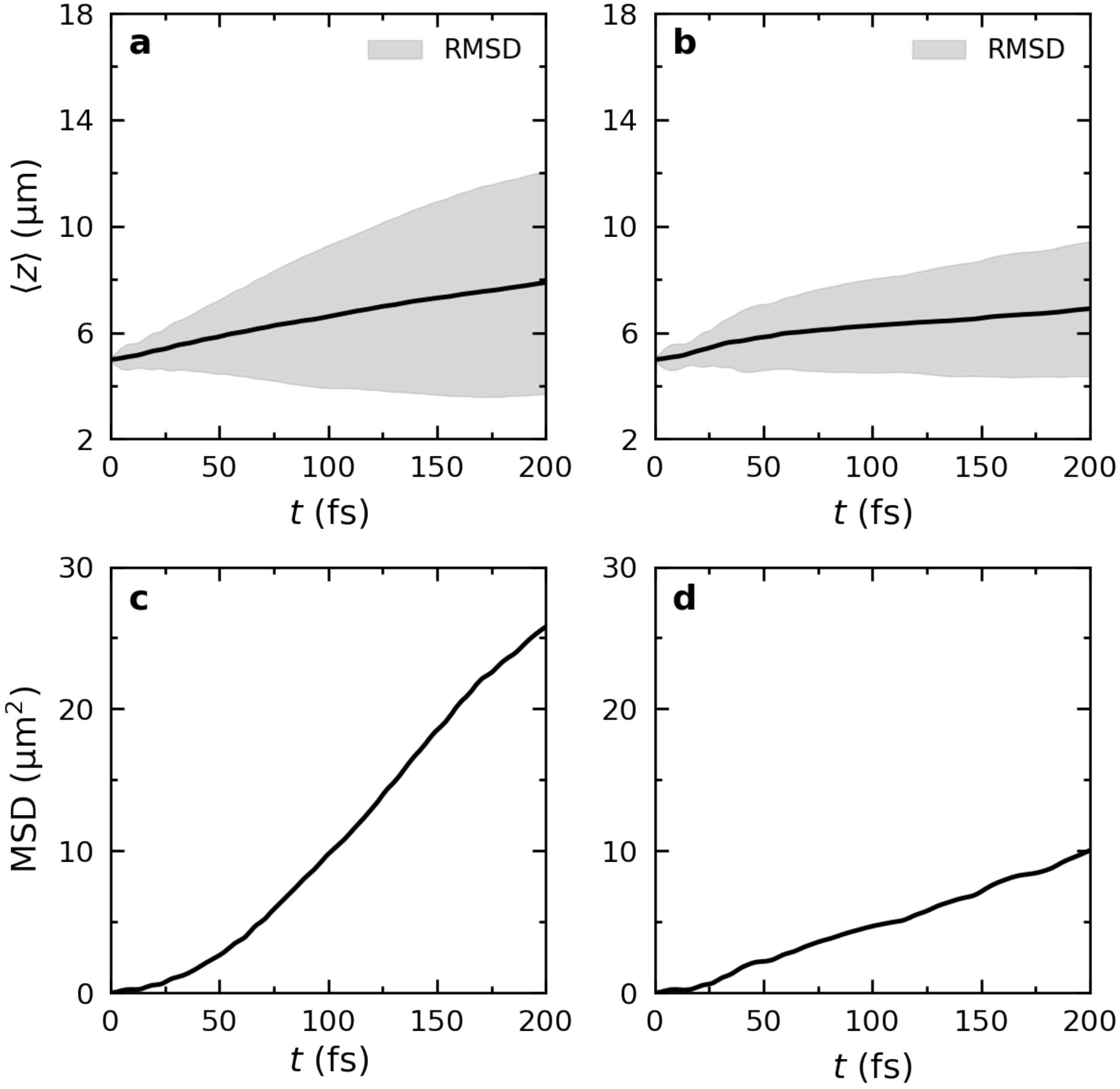

Supplementary Figure 13: Expectation value of the position ($\langle z \rangle = \frac{\langle \Psi(z,t)|\hat{z}|\Psi(z,t)\rangle}{\langle \Psi(z,t)|\Psi(z,t)\rangle}$, top panels) and mean squared displacement (MSD, *i.e.*, $\langle (z(t) - \langle z(0) \rangle)^2 \rangle$, bottom panels) of the total time-dependent wavefunction $|\Psi(z,t)\rangle$ of 512 Rhodamine molecules strongly coupled to an ideal cavity *i.e.*, $\gamma_{\mathrm{cav}} = 0$ ps^{-1} (panels **a** and **c**) and a lossy cavity with $\gamma_{\mathrm{cav}} = 66.7$ ps^{-1} (panels **b** and **d**), after off-resonant excitation of a single molecule. In the top panels, the black lines represents $\langle z \rangle$ while the shaded area shows the root mean squared deviation (RMSD, *i.e.*, $\sqrt{\langle (z(t) - \langle z(t) \rangle)^2 \rangle}$).

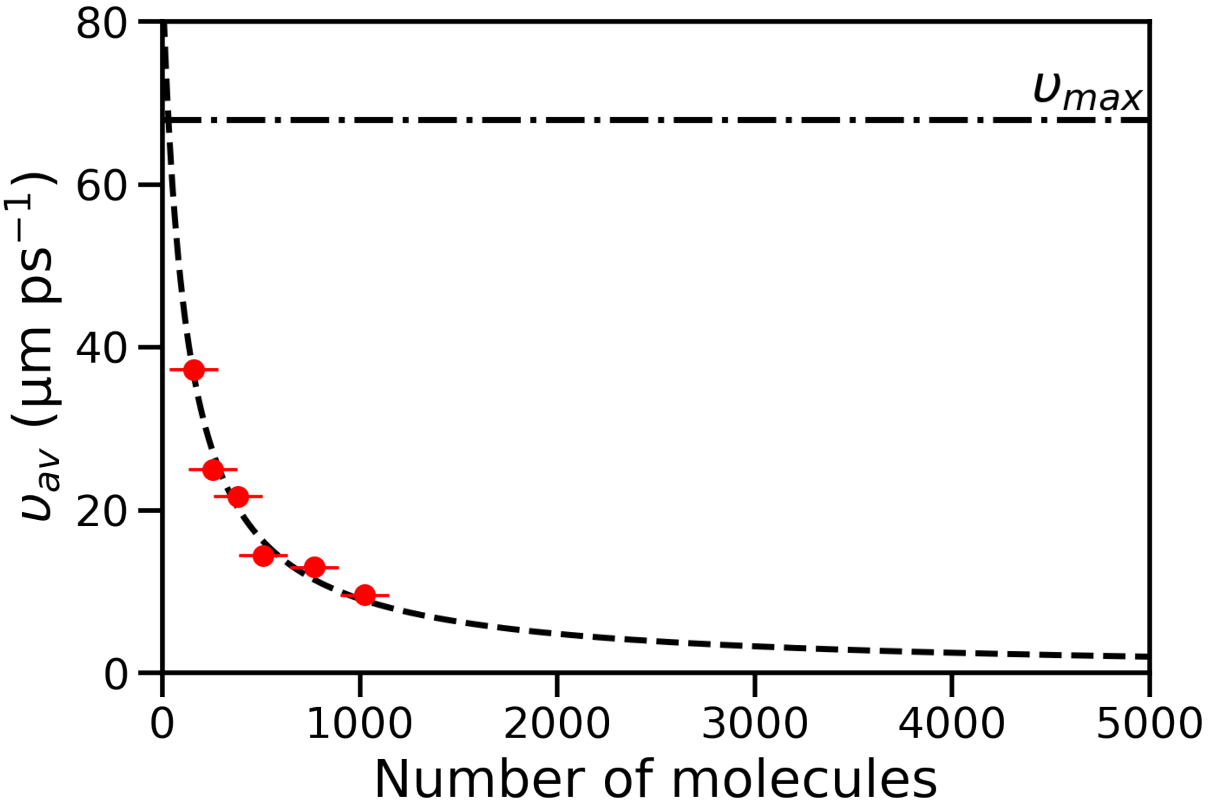

Supplementary Figure 14: Average propagation velocity υ_{av} as a function of number of molecules N in molecule-cavity systems after off-resonant excitation of a single molecule. Red dots are the values of υ_{av} extracted from the fits to the expectation value ($\langle z(t)\rangle$=$\langle\Psi(z,t)|\hat{z}|\Psi(z,t)\rangle/\langle\Psi(z,t)|\Psi(z,t)\rangle$) for different ensemble sizes. Red bars are the associated standard error calculated as a square root of the covariance of the fit parameter υ_{av} of the linear fit to $\langle z(t)\rangle$, *i.e.*, $\langle z(t)\rangle = \upsilon_{av}t$. The dashed black line shows the $a/\,(N+b)$ fit to $\upsilon_{\text{av}}(N)$ with $a = 10130$ μm ps^{-1} and $b = 117.24$. The dash-dotted line shows the value of the maximum group velocity $\upsilon_{\text{LP}}^{\text{max}}$ of the LP.

4.2 Comparison between Uni- and Bi-directional Cavity Models

In MD simulations of 1024 Rhodamine molecules strongly coupled to a symmetric cavity (*i.e.*, $n_{\text{min}} = -n_{\text{max}} = -80$) we observe that under off-resonant excitation conditions, the expectation value of the position operator, $\langle\Psi(t)|\hat{z}|\Psi(t)\rangle/\langle\Psi(t)|\Psi(t)\rangle$, remains zero in average, while the MSD increases approximately linearly (Supplementary Figure 15). The lack of net motion is due to cancellation between wavepackets propagating in opposite directions. Nevertheless, the excitation diffuses through the cavity as evidenced by the linear increase of the Mean Square Displacement (MSD), which is very similar to the MSD in the uni-directional cavity (Supplementary Figure 5**c**) that only included positive wavevectors.

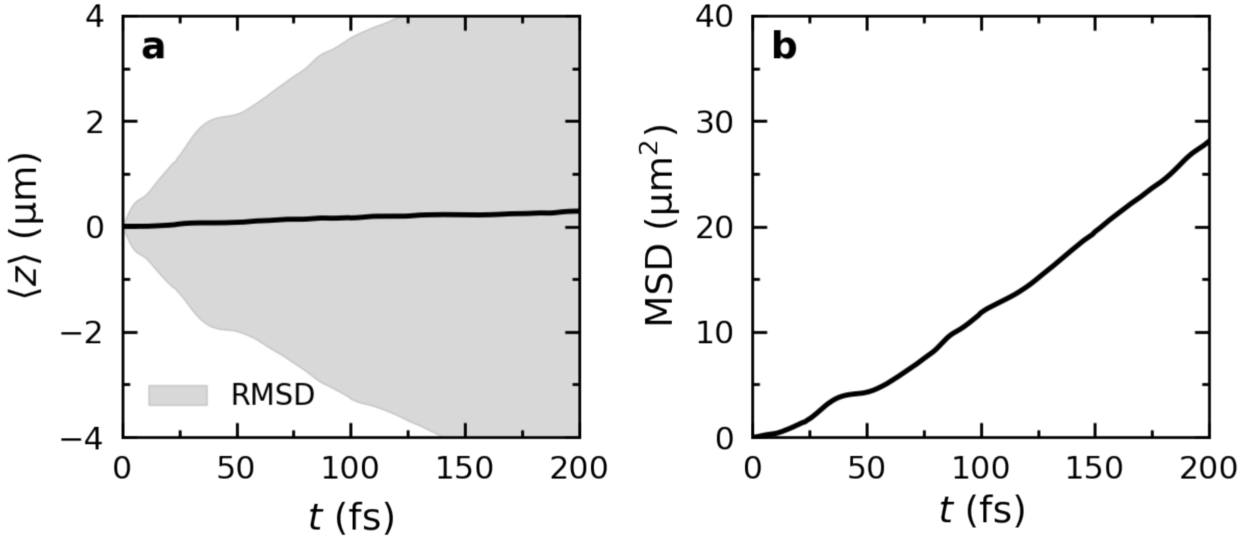

Supplementary Figure 15: (**a**) Expectation value of the position ($\langle z\rangle$=$\langle\Psi(z,t)|\hat{z}|\Psi(z,t)\rangle/\langle\Psi(z,t)|\Psi(z,t)\rangle$) and (**b**) mean square displacement (MSD, *i.e.*, $\langle(z(t)-\langle z(0)\rangle)^2\rangle$) of the total wavepacket $|\Psi(z,t)|^2$ of 1024 Rhodamine molecules strongly coupled to a symmetric, *i.e.*, bi-directional cavity without losses (*i.e.*, $\gamma_{\text{cav}} = 0$ ps^{-1}) after off-resonant excitation of a molecule located at 0 μm at 300 K.

4.3 Simulations at 0 K

We could previously show that population transfer between bright and dark states is mediated by nuclear dynamics.[47] Therefore, in *classical* MD simulations, a finite temperature is required for such non-adiabatic transitions. To understand the effect of the thermally activated nuclear dynamics on the transport, we repeated the simulations of 1024 Rhodamine molecules strongly coupled to the confined light modes of a cavity ($\omega_0 = 3.81$ eV, 160 modes) with perfect mirrors ($\gamma_{\text{cav}} = 0$ ps^{-1}) at 0 K. This hypothetical temperature was modelled by freezing all nuclear degrees of freedom.

4.3.1 On-resonant Excitation

In Supplementary Figure 16 we plot the probability density of the wavepacket as a function of time and position after on-resonant excitation at 0K. The wavepacket propagates ballistically at the central group velocity and retains its smooth Gaussian shape, in contrast to simulations at 300 K, in which peaks appear at the locations of molecules due to the fluctuations in their geometries and hence energies *i.e.* diagonal energy disorder.[8] Importantly, at 0K, no transition from ballistic to diffusive motion is observed (Supplementary Figure 17**d**), in line with experiments on inorganic materials at low temperatures.[49]

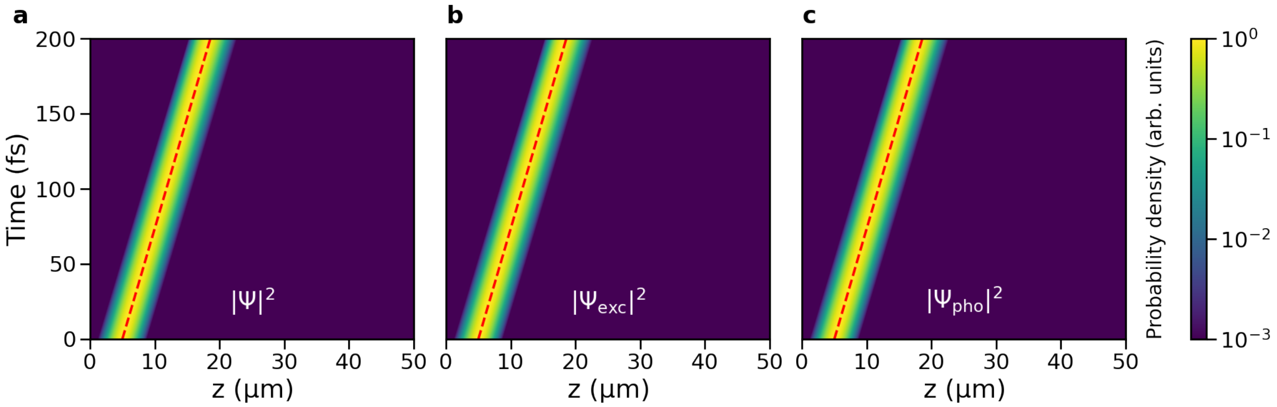

Supplementary Figure 16: Polariton propagation after on-resonant excitation of a wavepacket of lower polariton (LP) states centered at the k_z-vector corresponding to the maximum LP group velocity in a lossless cavity (*i.e.*, $\gamma_{\text{cav}} = 0$ ps^{-1}) containing 1024 Rhodamine molecules at 0 K. Panels **a**, **b** and **c**: total probability density $|\Psi(z,t)|^2$, probability density of the molecular excitons $|\Psi_{\text{exc}}(z,t)|^2$, and of the cavity mode excitations $|\Psi_{\text{pho}}(z,t)|^2$, respectively, as a function of distance (z, horizontal axis) and time (vertical axis). The red dashed line indicates propagation at the maximum group velocity of the LP (68 μm ps^{-1}).

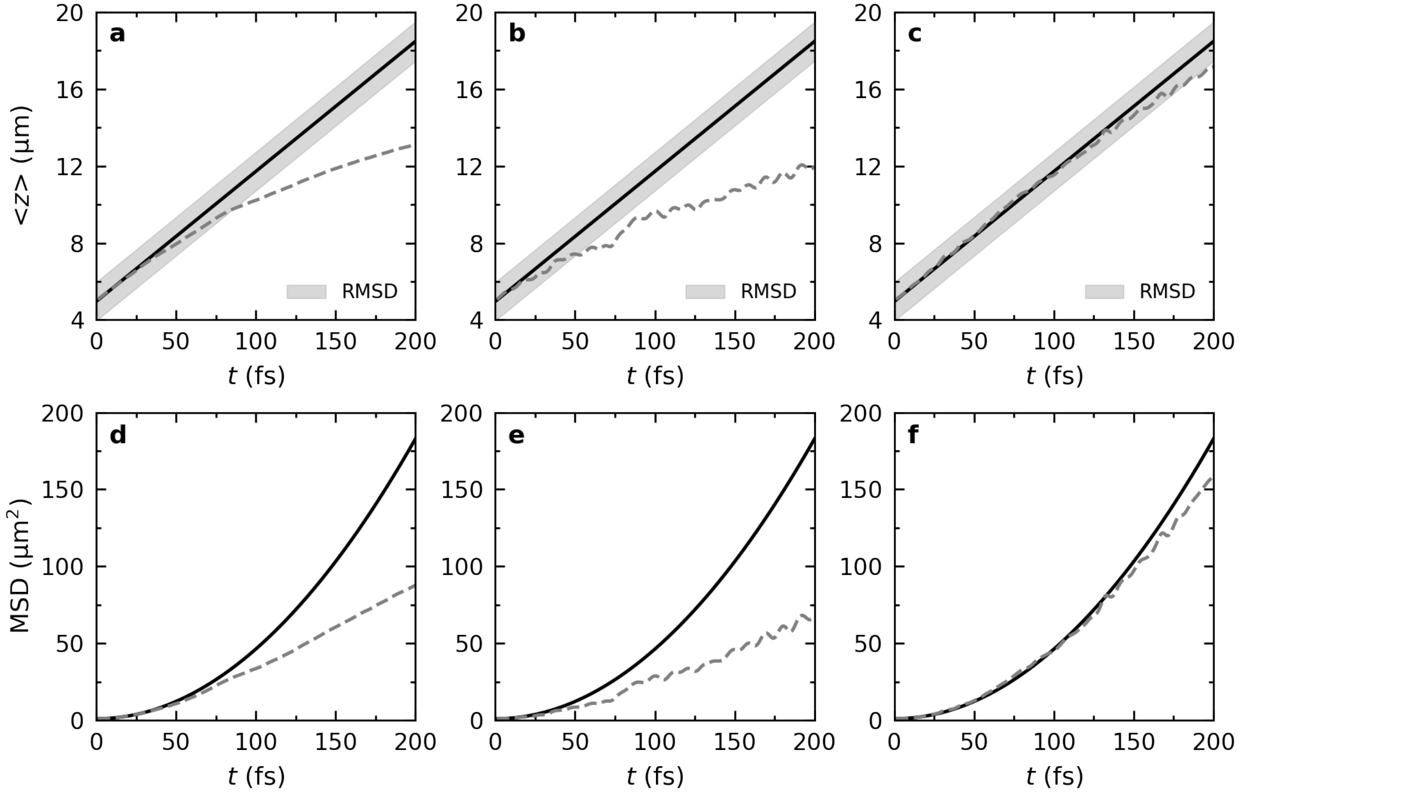

Supplementary Figure 17: Panels **a**, **b** and c: Expectation value of the position ($\langle z\rangle$=$\langle\Psi(z,t)|\hat{z}|\Psi(z,t)\rangle/\langle\Psi(z,t)|\Psi(z,t)\rangle$) of the total, $|\Psi(z,t)\rangle$, excitonic, $|\Psi_{\text{exc}}(z,t)\rangle$, and photonic, $|\Psi_{\text{pho}}(z,t)\rangle$, wave functions, repsectively, of 1024 Rhodamine molecules strongly coupled to a lossless cavity (*i.e.*, $\gamma_{\text{cav}} = 0$ ps^{-1}) after on-resonant excitation at 0 K. Panels **d**, **e** and f: Mean squared displacement (MSD, *i.e.*, $\langle(z(t) - \langle z(0)\rangle)^2\rangle$) of the total, $|\Psi(z,t)\rangle$ (left panels), excitonic, $|\Psi_{\text{exc}}(z,t)\rangle$ (middle panels), and photonic, $|\Psi_{\text{pho}}(z,t)\rangle$ (right panels), wave functions, respectively, of 1024 Rhodamine molecules strongly coupled to a lossless cavity (*i.e.*, $\gamma_{\text{cav}} = 0$ ps^{-1}) after on-resonant excitation at 0 K. As a reference, the curves corresponding to the same system at 300 K are plotted as grey dashed lines. The shaded area around the lines corresponding to $\langle z\rangle$ at 0 K represents the root mean squared deviation (RMSD, *i.e.*, $\sqrt{\langle(z(t) - \langle z(t)\rangle)^2\rangle}$).

4.3.2 Off-resonant Excitation

In Supplementary Figure 18 we plot the probability density of the wavepacket as a function of time and position after off-resonant excitation at 0K. Compared to the time-evolution of the wavepacket at 300 K, the propagation is reduced at 0K, and most of the excitation remains localized at the molecule that was initially excited (Supplementary Figure 18**b**). The reduced mobility of the *total* wavepacket is visible also in the evolution of the expectation value of $\langle z \rangle$ in Supplementary Figure 19**a**. In contrast to the total wavepacket and the contribution of the molecular excitons, the part of the wavepacket that consists of the cavity mode excitations propagates ballistically (Supplementary Figure 19**c** and **f**). Thus, while most of the wavepacket remains localized on the molecule that was excited initially, a small fraction of bright states propagate ballistically. Because there are no nuclear dynamics at 0K, this propagation is solely driven by constructive and destructive interferences between bright states that were occupied in the initial superposition, and that are due to differences in their phases (*i.e.*, Rabi oscillations: $c_m(t) = c_m(0)e^{-iE_m t/\hbar}$).

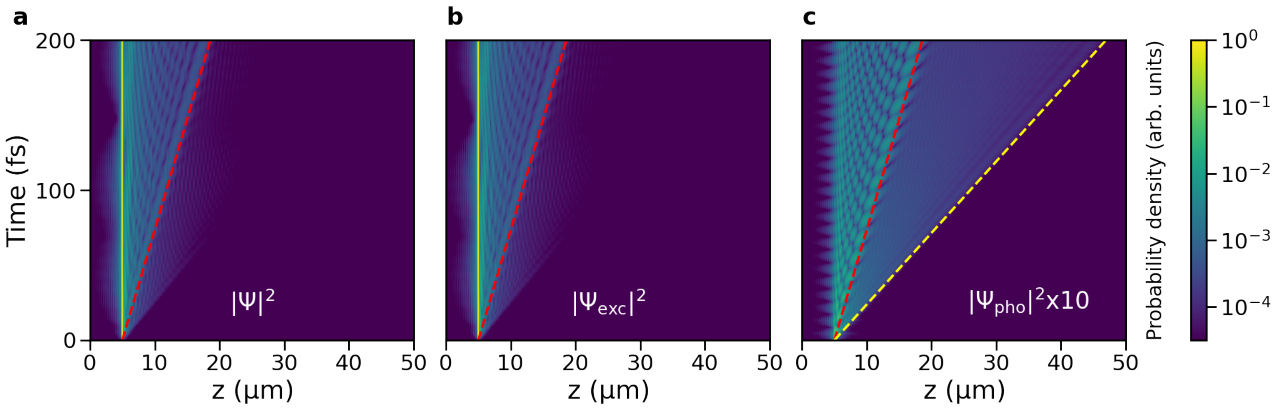

Supplementary Figure 18: Polariton propagation after off-resonant excitation of a single molecule located at $z = 5$ μm into S_1 state at 0 K. Panels **a**, **b** and **c**: total probability density $|\Psi(z,t)|^2$, probability density of the molecular excitons $|\Psi_{\text{exc}}(z,t)|^2$, and of the cavity mode excitations $|\Psi_{\text{pho}}(z,t)|^2$, respectively, as a function of distance (z, horizontal axis) and time (vertical axis), in a cavity with perfect mirrors (*i.e.*, $\gamma_{\text{cav}} = 0$ ps^{-1}). The red and yellow dashed lines indicate propagation at the maximum group velocity of the lower polaritons (68 μm ps^{-1}) and upper polaritons (212 μmps^{-1}), respectively.

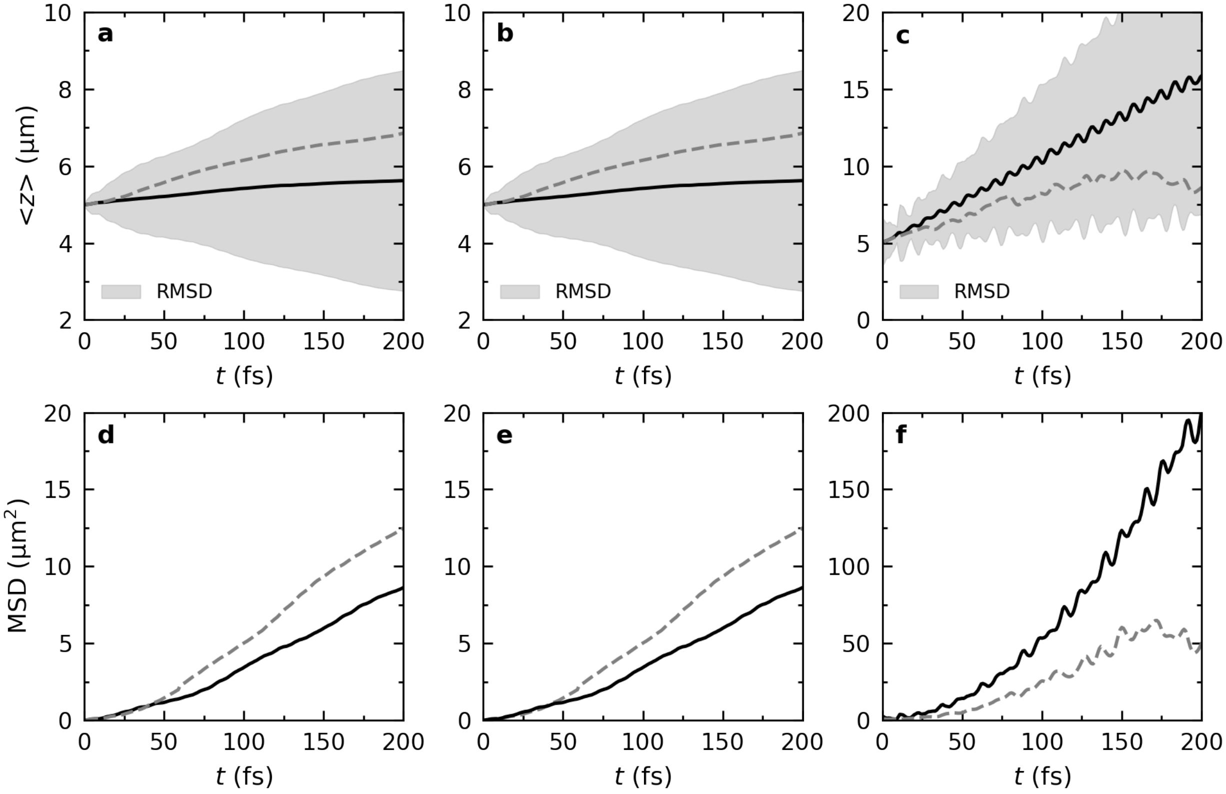

Supplementary Figure 19: Panels **a**, **b** and **c**: Expectation value of the position ($\langle z\rangle$=$\langle\Psi(z,t)|\hat{z}|\Psi(z,t)\rangle/\langle\Psi(z,t)|\Psi(z,t)\rangle$) of the total, $|\Psi(z,t)\rangle$ (left panels), excitonic, $|\Psi_{\text{exc}}(z,t)\rangle$ (middle panels), and photonic, $|\Psi_{\text{pho}}(z,t)\rangle$ (right panels), wave functions, respectively, of 1024 Rhodamine molecules strongly coupled to a lossless cavity (*i.e.*, $\gamma_{\text{cav}} = 0$ ps^{-1}) after off-resonant excitation at 0 K. Panels **d**, **e** and **f**: mean squared displacement (MSD, *i.e.*, $\langle(z(t) - \langle z(0)\rangle)^2\rangle$, bottom panels) of the total, $|\Psi(z,t)\rangle$ (left panels), excitonic, $|\Psi_{\text{exc}}(z,t)\rangle$ (middle panels), and photonic, $|\Psi_{\text{pho}}(z,t)\rangle$ (right panels), wave functions, respectively, of 1024 Rhodamine molecules strongly coupled to a lossless cavity (*i.e.*, $\gamma_{\text{cav}} = 0$ ps^{-1}) after off-resonant excitation at 0 K. As a reference, the curves corresponding to the same system at 300 K are plotted as grey dashed lines. Note that we plot both $\langle z\rangle$ and MSD only until $t = 200$ fs, since at later times the part of the wave packet composed of upper polariton states has crossed the periodic boundary at $z = 50$ μm. The shaded area around the lines corresponding to $\langle z\rangle$ at 0 K represents the root mean squared deviation (RMSD, *i.e.*, $\sqrt{\langle(z(t) - \langle z(t)\rangle)^2\rangle}$).

4.4 Non-Adiabatic Transitions

As shown in the previous section, polariton propagation at 300 K differs significantly from motion at 0K. To understand if these differences solely arise from dynamically induced disorder or also involve non-adiabatic transitions, we plot the time-evolution of the expansion coefficients of the adiabatic LP, UP and dark states (*i.e.*, $\sum_m |c_m(t)|^2$ with $m \in$ LP (160 low-energy states), UP (160 higher-energy states), and DS (the remaining states), respectively) in Supplementary Figure 20 for 256 molecules in an ideal lossless cavity after on-resonant and off-resonant excitation. The variation in the magnitude of these coefficients with time suggest that there is significant non-adiabatic population transfer between bright and dark states in our simulations.

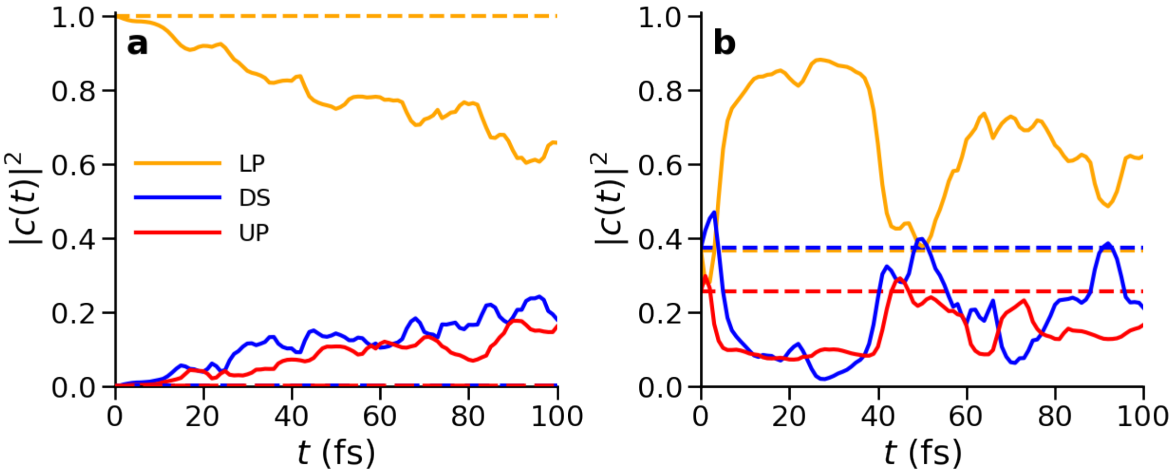

Supplementary Figure 20: Time-evolution of the sum of expansion coefficients $\sum_m |c_m(t)|^2$ of states belonging to the LP (orange line), the UP (red line) and the dark states manifold (blue line) at 300 K (solid lines) and at 0K (dashed lines) for the system with 256 Rhodamine molecules strongly coupled to an ideal cavity (*i.e.*, $\gamma_{\text{cav}} = 0$ ps^{-1}) after off-resonant (panel **a**) and on-resonant (panel **b**) excitation.

4.5 Effect of Positional Disorder on Polariton Propagation

In our simulations the solvated Rhodamine molecules were located equidistantly along the z-axis of the 1D cavity. To verify if such ordering affects the propagation process, we repeated a simulation under off-resonant excitation conditions with 512 Rhodamines *randomly* distributed along the z-axis of the cavity. This was achieved by adding a random displacement to the initial equidistant positions. We only considered a perfect cavity with no losses ($\gamma_{\text{cav}} = 0$ ps^{-1}) and with a vacuum field strength of 0.0000707107 au (0.364 MVcm^{-1}), yielding a Rabi splitting of $\sim$ 325 meV. Direct visual comparison between the wavepacket dynamics in this system, shown in Supplementary Figure 21, to that in the system with an equal spacing between the molecules (third row in Supplementary Figure 9), suggest that positional disorder does not influence polariton propagation, nor the time evolution of the wave function composition (Supplementary Figure 21**d** and Supplementary Figure 9**h**).

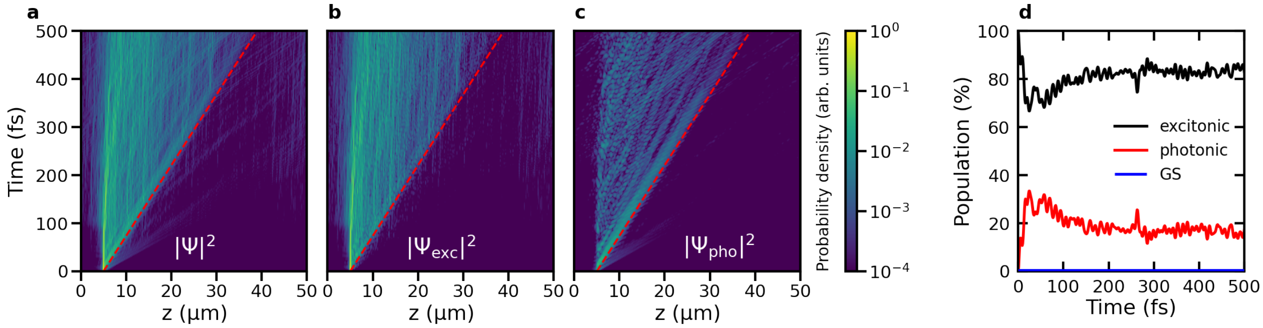

Supplementary Figure 21: Polariton propagation after off-resonant excitation into the S_1 state of a single molecule located at $z = 5$ μm, in an ensemble of 512 Rhodamine molecules randomly distributed along the z-axis of an ideal cavity (*i.e.*, $\gamma_{\text{cav}} = 0$ ps^{-1}). Panels **a**, **b** and **c**: total probability density, $|\Psi(z,t)|^2$, probability density of the molecular excitons, $|\Psi_{\text{exc}}(z,t)|^2$, and of the cavity mode excitations, $|\Psi_{\text{pho}}(z,t)|^2$, respectively, as a function of distance (z, horizontal axis) and time (vertical axis). Panel **d**: Contributions of molecular excitons (black) and cavity mode excitations (red) to $|\Psi(z,t)|^2$ and population of the ground state (GS, blue) as a function of time.

4.6 Effect of Initial Energy Disorder on Ballistic Transport

Starting simulations with the same molecular geometries, and thus identical excitation energies and transition dipole moments, facilitates the identification of bright and dark states. However, in experiments the molecular geometries span a distribution weighted by their Boltzmann factors. To assess the effect of such initial (thermal) disorder on polariton propagation after resonant excitation, we repeated a simulation of 256 Rhodamines inside a lossless cavity ($\gamma_{\text{cav}} = 0$ ps^{-1}) with a vacuum field strength of $\mathbf{E}_y = 0.0001$ a.u. (0.514 MVcm^{-1}), with different initial geometries and velocities. These configurations were selected from the equilibrium QM/MM trajectory described in section 2.

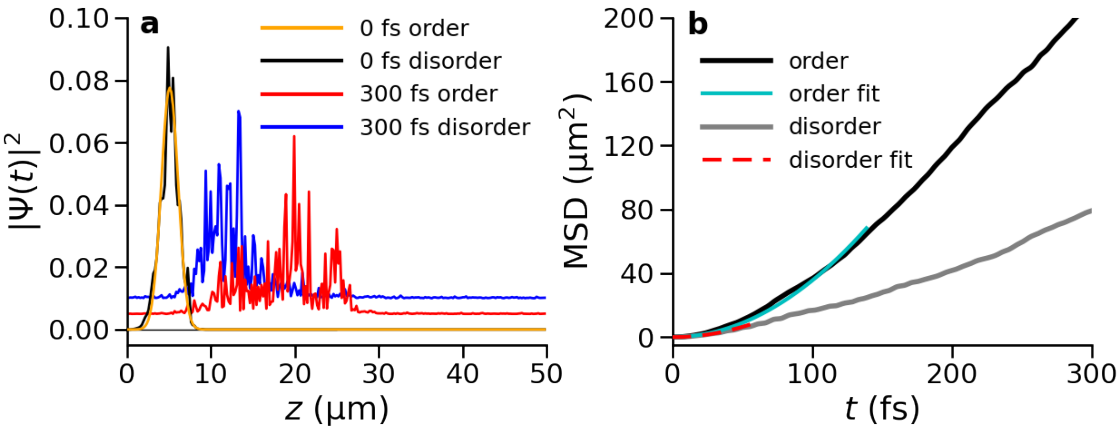

Supplementary Figure 22: Panel **a**: Total probability density, $\left|\Psi(z,t)\right|^2$, of 256 Rhodamine molecules strongly coupled to an ideal cavity (*i.e.*, $\gamma_{\text{cav}} = 0$ ps^{-1}) with (black line at $t = 0$ fs and blue line at $t = 300$ fs) and without (orange line at $t = 0$ fs and red line at $t = 300$ fs) initial energy disorder after on-resonant excitation of lower polaritons. Panel **b**: Mean squared displacement (MSD) of the total wave function, $|\Psi(z,t)\rangle$, with (grey) and without (black) initial geometric disorder among the molecules. The cyan and red dashed lines are quadratic fits to the MSD in the ballistic phase.

As shown in panel **a** of Supplementary Figure 22, with energetic disorder, the initial wavepacket is no longer a smooth Gaussian curve (green line), but its propagation is very similar to that of a wavepacket starting without such disorder (black line): Initially, there is ballistic motion at a velocity close to the maximum group velocity of the LP, followed by diffusion (see Supplementary Figure 22**b**). However, because with the initial energy disorder non-adiabatic population transfers into lower energy dark states occur more readily from the start of the simulation, the transition

between ballistic and diffusive transport happens earlier.

4.7 Effect of Rabi Splitting on Polariton Propagation

The results of our MD simulations suggest that reversible population transfers between bright polaritonic states with group velocity and dark states without group velocity, play an important role in the propagation of a polaritonic wavepacket. Because the non-adiabatic coupling vectors that control these population transfers are inversely proportional to the energy gap between the states,[47] altering the overlap between dark and bright states by changing the Rabi splitting could impact polariton propagation. To investigate how the Rabi splitting affects the propagation of the polariton wavepacket, we repeated simulations for 1024 molecules strongly coupled to the confined light modes of a lossy cavity ($\gamma_{\text{cav}} = 66.7$ ps^{-1}) after on-resonant and off-resonant excitation at three different Rabi splittings. In these simulations, the timestep of the MD simulations was increased to 0.5 fs. To alter the Rabi splitting without changing the number of states, we varied the vacuum field strengths: $|\mathbf{E}_y| = 0.000025$, 0.000037 and 0.00005 a.u., yielding Rabi splittings of $\sim$ 159, 240 and 325 meV. We carried out two simulations per Rabi splitting value with different initial velocities. To facilitate direct comparison, the same random velocities were used for each Rabi splitting. The latter was realized by using the index of the molecule as a random seed for selecting the velocities in the first series of simulations, and adding 1024 to that index in the second series.

4.7.1 On-resonant Excitation

In Supplementary Figure 23a we show the Mean Square Displacement (MSD) of the total wave function for various Rabi splitting energies after on-resonant excitation of a wavepacket of LP states. While the initial velocity in the ballistic phase reflects the central group velocity of the wavepacket, which is highest for the smallest Rabi splitting (81 μm/ps *vs.* 68 μm/ps for the largest Rabi splittings), there is no clear trend at longer timescales, suggesting that more simulations are needed.

Based on previous results, which suggest that increasing the vacuum field strength decreases the

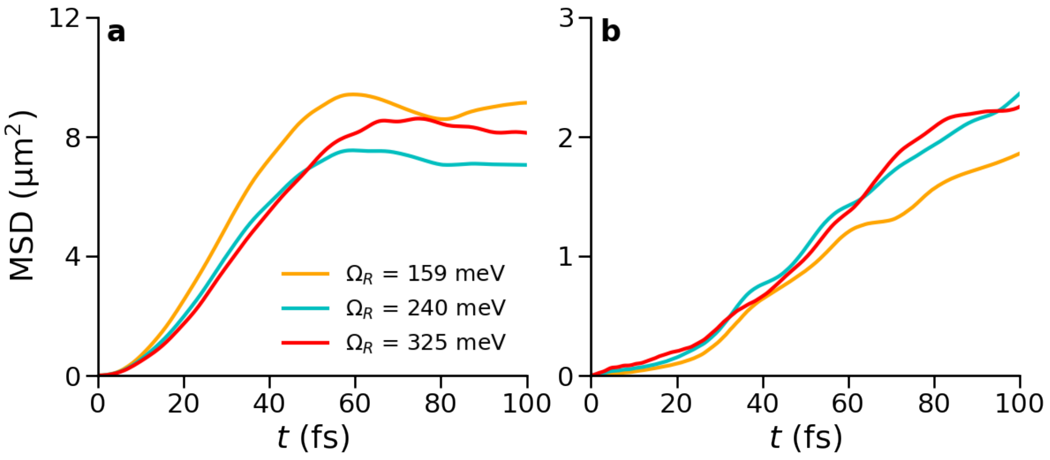

Supplementary Figure 23: Effect of Rabi splitting on the Mean Squared Displacement (MSD) of the total polariton wave function, $|\Psi(z,t)\rangle$, after on-resonant (panel **a**) and off-resonant (panel **b**) excitation into the lower polariton branch of a system with 1024 molecules strongly coupled to a lossy cavity ($\gamma_{\text{cav}} = 66.7$ ps^{-1}).

overlap between the bright polaritonic branches and dark state manifold,[21] we speculate that the decrease in group velocity upon increasing the Rabi splitting, may be compensated by a reduction of the population transfer into the dark states. To minimize that population transfer and extend the ballistic phase, the Rabi splitting should exceed the absorption line width of the molecules,[21] which for typical organic materials would require entering the ultra-strong coupling regime, where the Rabi splitting is more than 10% of the excitation energy.[1] However, in this regime, the Tavis-Cummings models is no longer strictly valid as the RWA breaks down, and the counter-rotating terms ($\hat{\sigma}_j^+ \hat{a}_{k_z}^\dagger$ and $\hat{\sigma}_j \hat{a}_{k_z}$), as well as the dipole self energy ($\hat{H}^{\text{DSE}} = \frac{1}{2\epsilon_0 V_{\text{cav}}} \left[\mathbf{u}_{\text{y}} \cdot \left(\sum_i^N \hat{\boldsymbol{\mu}}_i\right)\right]^2$) would need to be included,[50] which is computationally intractable for the system sizes used in this work.

Nevertheless, even if our results do not reveal a clear trend, there may be a trade-off between the absorption line width of the material and the Rabi splitting, for optimal exciton transport. However, because the focus of the current work is on the *mechanism* of polariton propagation, we consider finding that trade-off beyond the scope of our work, and will address the effect of the Rabi splitting on polariton transport in future work.

4.7.2 Off-resonant Excitation

In Supplementary Figure 23b we show the Mean Square Displacement of the total wavepacket for the various Rabi splittings after off-resonant excitation of a single molecule located at $z = 5$ µm. As for on-resonance excitation, we do not observe a clear correlation between the Rabi splitting on the one hand and the Mean Square Displacement on the other hand. Very many additional simulations are probably needed to extract a trend. However, because the focus of the current work is on the *mechanism* of polariton propagation, we consider a thorough investigation of the effect of the Rabi splitting beyond the scope of the current work. Yet, since we anticipate that the Rabi splitting may have an effect that could possibly be exploited and can be directly controlled via the molecular concentration, we will address the effect of the concentration on polariton transport in future work.

4.8 Contribution of High-Energy Polariton States to the Wavepacket Propagation

Following *off-resonant* excitation into the S_1 electronic state of a single Rhodamine molecule, we observe that the photonic part of the wavepacket has a small front that propagates faster than the rest of the wavepacket. In Supplementary Figure 24 we show the probability density of the cavity mode excitations in the 512 molecule cavity system at 50 and 100 fs. The wavepacket is composed of a slower and faster component, with the front of the faster component reaching distances corresponding to the maximum group velocity $v_{\mathrm{UP}}^{\mathrm{max}}$ = 212 μmps^{-1} of the UP branch.

To confirm that the fastest components in the wavepacket are due to propagation of population in the UP states that get rapidly populated due to Rabi oscillations (Equation 24), we analyzed the photoluminesce through 10 μm wide pinholes located at z = 10 μm and at z = 20 μm (see section 3.5 for details of these calculations).[37] The photoluminescence spectra collected through these pinholes are plotted as a function of the in-plane momentum (k_z) in Figures 24**b** and **c**. While at z = 10 μm the photoluminescence is due to emission from both LP and UP states, emission through the pinhole at z = 20 μm originates exclusively from UP states. These observations thus confirm that the fastest moving part of the wave packet is composed of UP states.

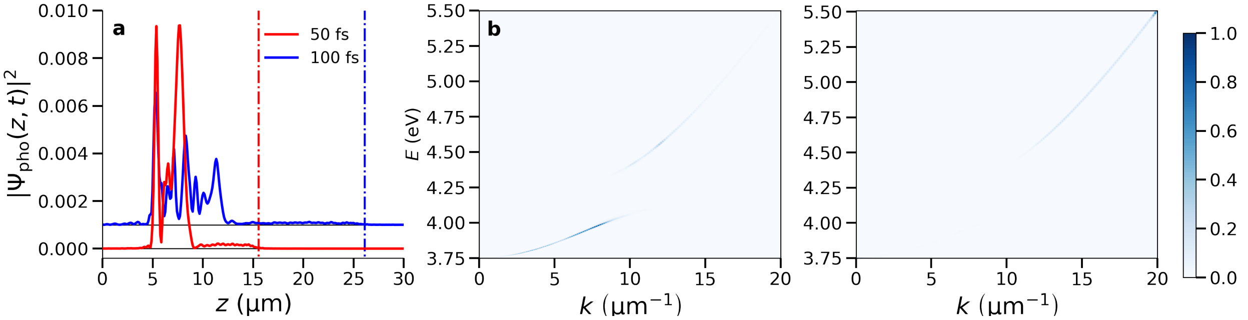

Supplementary Figure 24: Panel **a**: Probability densities of $\left|\Psi_{\mathrm{pho}}(z,t)\right|^2$ at $t = 50$ and 100 fs after off-resonant excitation of a cavity including 512 Rhodamine molecules. The initial excitation is localised on molecule placed at $z = 5$ μm. The dashed-dotted lines indicate the product between the maximum group velocity of the upper polaritons ($v_{\mathrm{UP}}^{\mathrm{max}}$ = 212 μmps^{-1}) and the simulation time, and thus correspond to where something moving at that group velocity would be located. Panels **b** and **c**: Photoluminescence spectra collected through pinholes located at $z = 10$ μm and $z = 20$ μm, respectively.

4.9 Polariton Propagation in Tetracene and Methylene Blue cavities

With an absorption linewidth of 100 meV (FWHM), a Stokes shift of 120 meV, and a minimum energy conical intersection sufficiently above the Franck Condon region to prevent internal conversion (1.3 eV), our Rhodamine model captures the key photophysical features of typical organic dye molecules. Therefore, we consider the propagation mechanism observed in our simulations, representative for organic polaritons in general. Nevertheless, to demonstrate that the mechanism is robust with respect to changes in the optical properties of the molecules, we repeated the simulations for a cavity filled with 1024 Tetracene molecules in cyclohexane, and for a cavity with 1024 Methylene Blue dyes in water. At the CIS/3-21G level of theory employed in our simulations, Tetracene has an absorption line width of 0.14 eV and a Stokes shift of 0.7 eV, both of which are significantly higher than for Rhodamine. The Rabi splitting of the Tetracene cavity was 0.24 eV. At the TD-B97/3-21G level of theory, Methylene blue has an absorption line width of 0.07 eV and a Stokes shift of 0.23 eV. The Rabi splitting of the Methylene Blue cavity was 0.175 eV.

4.9.1 Tetracene

In Supplementary Figure 25 we show polariton propagation in a cavity filled with Tetracene in cyclohexane following an on-resonant excitation of a wavepacket of LP states for both a lossless ($\gamma_{\text{cav}} = 0$ ps^{-1}) and a lossy cavity ($\gamma_{\text{cav}} = 66.7$ ps^{-1}). After a very short ballistic phase, which is only discernible as the steep initial rise of $\langle z \rangle$ in the top panels of Supplementary Figure 26, propagation becomes diffusive, as evidenced by a linear time-dependence of the Mean Square Displacement (Supplementary Figure 26**c** and **d** for the lossless and lossy cavity, respectively). The absence of a clear coherent transport regime is due to the large absorption linewidth of Tetracene. As we could show previously,[21] the rate at which population transfers from the LP into the dark states depends on the energetic overlap between the LP and the molecular density of states, with the latter matching the absorption spectrum). Because for Tetracene the absorption spectrum is much broader than the Rabi splitting, this overlap is very large and hence the population transfer is very fast. As a consequence, the excitonic contributions start dominating the wavepacket immediately after the

excitation, ending the ballistic propagation phase earlier than in cavities filled with Rhodamine, for which the overlap between the LP and the molecular dark states is much smaller. Because these transfers are reversible, the propagation continues in a diffusive manner. Adding losses does not change this picture, but because the coherent phase is much shorter than in cavities filled with Rhodamine, also the total radiative decay is smaller (bottom rows in Figures 2 and 25).

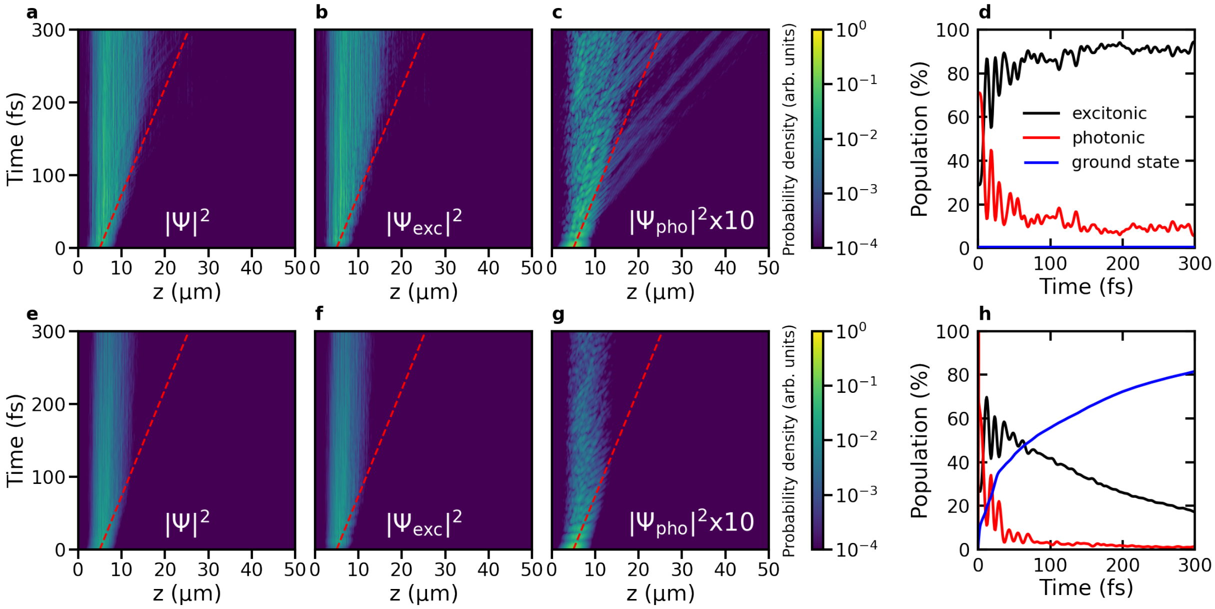

Supplementary Figure 25: Polariton propagation in cavities filled with Tetracene molecules after on-resonant excitation of a wavepacket of lower polariton (LP) states, centered at $z = 5$ μm. Panels **a**, **b** and **c**: total probability density $|\Psi(z,t)|^2$, probability density of the molecular excitons $|\Psi_{\text{exc}}(z,t)|^2$ and of the cavity mode excitations $|\Psi_{\text{pho}}(z,t)|^2$, respectively, as a function of distance (z, horizontal axis) and time (vertical axis), in a cavity with perfect mirrors (*i.e.*, $\gamma_{\text{cav}} = 0$ ps^{-1}). The red dashed line indicates propagation at the maximum group velocity of the LP (68 μmps^{-1}). Panel **d**: Contributions of molecular excitons (black) and cavity mode excitations (red) to $|\Psi(z,t)|^2$ as a function of time in the perfect cavity. With no photon losses, no ground state population (blue) can build up. Panels **e**, **f**, and **g**: $|\Psi(z,t)|^2$, $|\Psi_{\text{exc}}(z,t)|^2$ and $|\Psi_{\text{pho}}(z,t)|^2$, respectively, as a function of distance (z, horizontal axis) and time (vertical axis), in a lossy cavity (*i.e.*, $\gamma_{\text{cav}} = 66.7$ ps^{-1}). Panel **h**: Contributions of the molecular excitons (black) and cavity mode excitations (red) to $|\Psi(z,t)|^2$ as a function of time. The population in the ground state, created by radiative decay through the imperfect mirrors, is plotted in blue.

In Supplementary Figure 27 we show the wavepacket dynamics in both a lossless ($\gamma_{\text{cav}} = 0$ ps^{-1}) and a lossy cavity ($\gamma_{\text{cav}} = 66.7$ ps^{-1}) following off-resonant excitation of the Tetracene molecule located at 5 μm. As in cavities filled with Rhodamine (Supplementary Figure 3 in the main text),

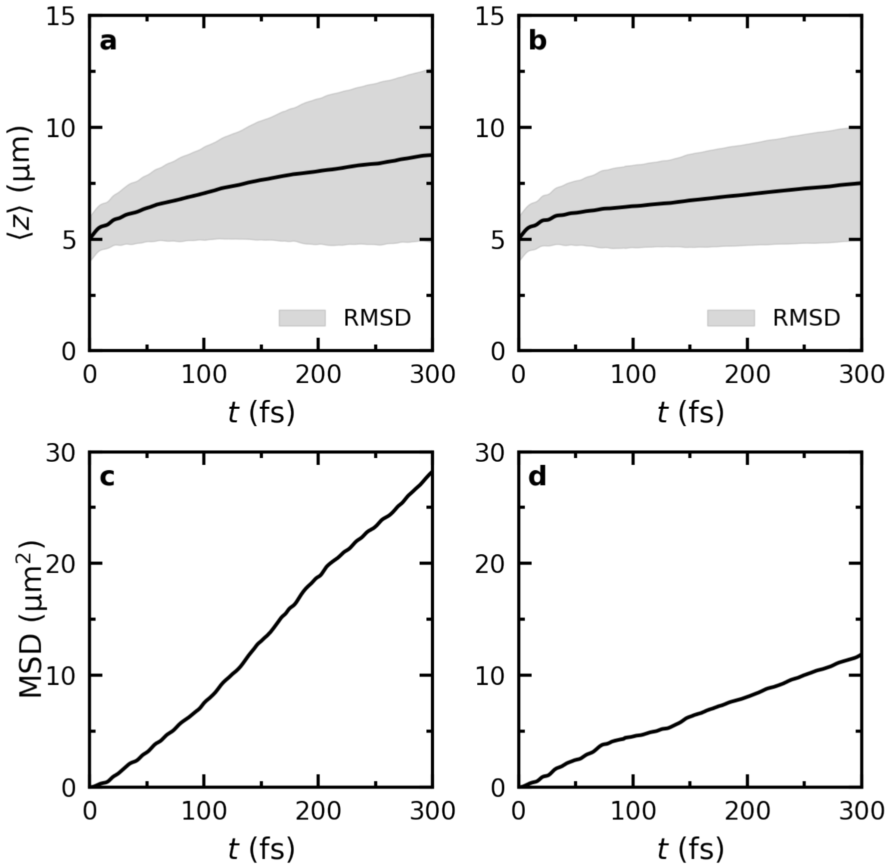

Supplementary Figure 26: Top panels: Expectation value of the position of the total time-dependent wavefunction $\langle z\rangle$=$\langle\Psi(z,t)|\hat{z}|\Psi(z,t)\rangle/\langle\Psi(z,t)|\Psi(z,t)\rangle$ after on-resonant excitation of lower polariton (LP) states in lossless (**a**, $\gamma_{\text{cav}} = 0$ ps^{-1}) and lossy cavities (**b**, $\gamma_{\text{cav}} = 66.7$ ps^{-1}) filled with Tetracene molecules. The black lines represent $\langle z\rangle$ while the shaded area around the lines represents the root mean squared deviation (RMSD, *i.e.*, $\sqrt{\langle(z(t)-\langle z(t)\rangle)^2\rangle}$). Bottom panels: Mean Squared Displacement (MSD, *i.e.*, $\langle(z(t)-\langle z(0)\rangle)^2\rangle$) in the ideal lossless cavity (**c**) and the lossy cavity (**d**).

propagation is diffusive, driven by reversible exchange of population between bright states with group velocity and dark states without group velocity. Because for the on-resonant excitation, the initial coherent ballistic phases is very short due to the broad absorption of Tetracene strongly overlapping with the LP branch, the Mean Square Displacements are similar for both excitation schemes, in particular for the lossless cavity (Figures 26 and Figures 28). This is in contrast to cavities filled with Rhodamine for which the longer lasting coherent phase with ballistic propagation, leads to a faster initial rise of the Mean Square Displacement, until the turn-over into the diffusion regime occurs (Supplementary Figure 2 in the main text).

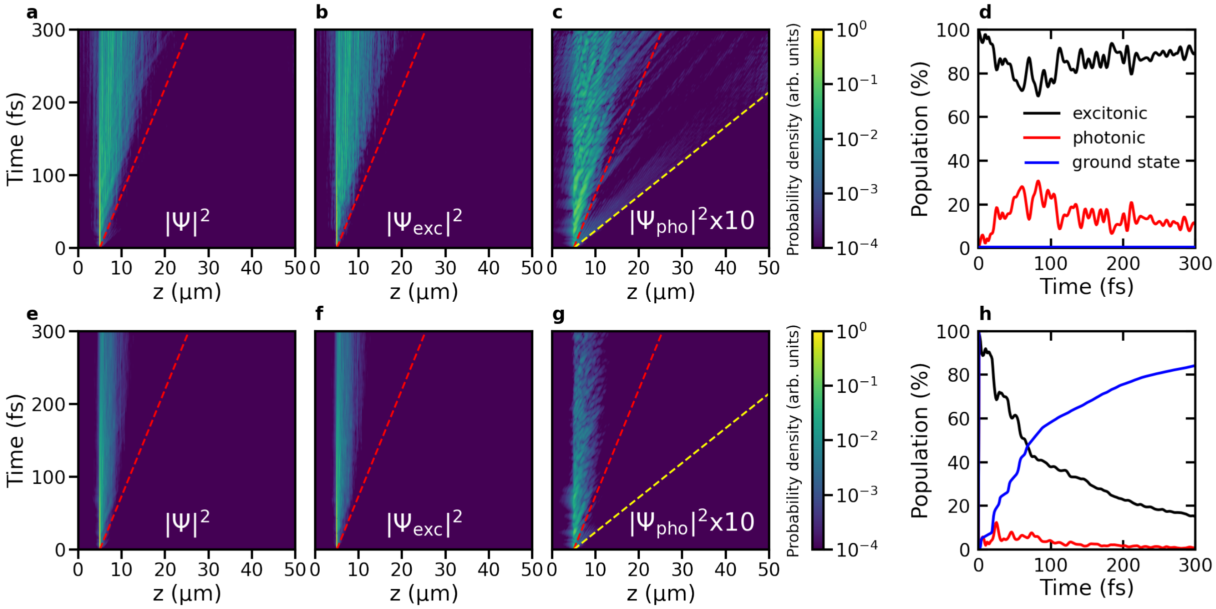

Supplementary Figure 27: Polariton propagation in cavities filled with Tetracene molecules after off-resonant excitation of a molecule located at $z = 5$ μm. Panels **a**, **b** and **c**: total probability density $|\Psi(z,t)|^2$, probability density of the molecular excitons $|\Psi_{\rm exc}(z,t)|^2$ and of the cavity mode excitations $|\Psi_{\rm pho}(z,t)|^2$, respectively, as a function of distance (z, horizontal axis) and time (vertical axis), in a cavity with perfect mirrors (*i.e.*, $\gamma_{\rm cav} = 0$ ps^{-1}). The red and yellow dashed lines indicate propagation at the maximum group velocity of the lower polaritons (68 μmps^{-1}) and upper polaritons (220 μmps^{-1}), respectively. Panel **d**: Contributions of molecular excitons (black) and cavity mode excitations (red) to $|\Psi(z,t)|^2$ as a function of time in the perfect cavity. With no photon losses, no ground state population (blue) can build up. Panels **e**, **f**, and **g**: $|\Psi(z,t)|^2$, $|\Psi_{\rm exc}(z,t)|^2$ and $|\Psi_{\rm pho}(z,t)|^2$, respectively, as a function of distance (z, horizontal axis) and time (vertical axis), in a lossy cavity (*i.e.*, $\gamma_{\rm cav} = 66.7$ ps^{-1}) Panel **h**: Contributions of the molecular excitons (black) and cavity mode excitations (red) to $|\Psi(z,t)|^2$ as a function of time. The population in the ground state, created by radiative decay through the imperfect mirrors, is plotted in blue.

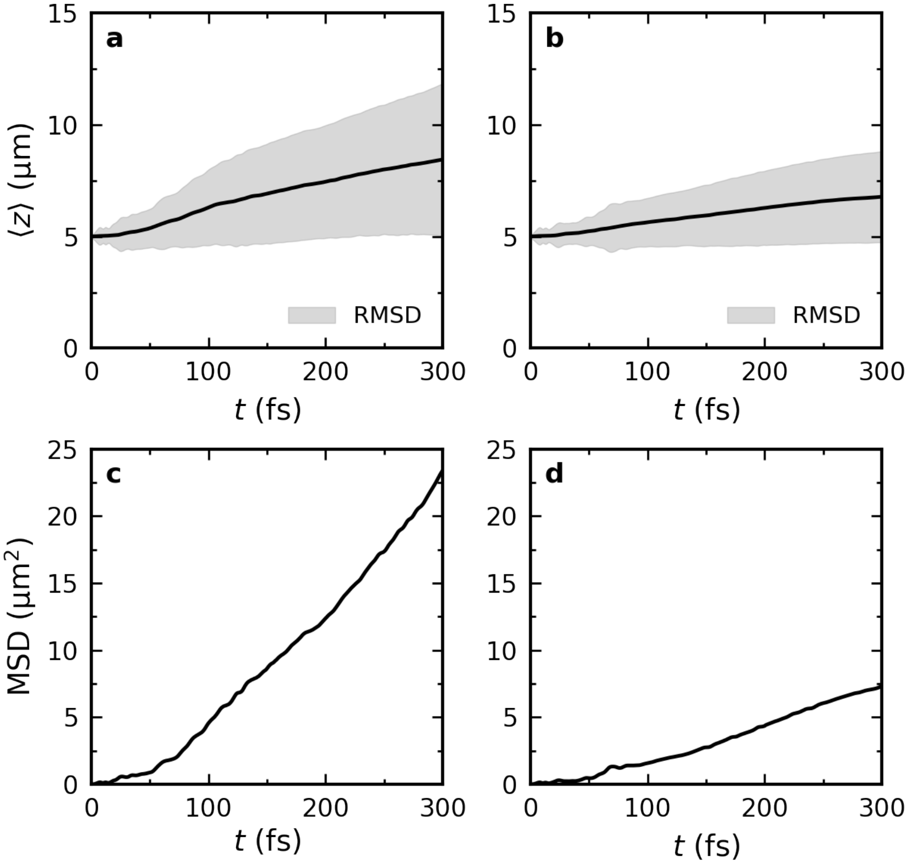

Supplementary Figure 28: Top panels: Expectation value of the position of the total-time dependent wavefunction $\langle z\rangle$=$\langle\Psi(z,t)|\hat{z}|\Psi(z,t)\rangle/\langle\Psi(z,t)|\Psi(z,t)\rangle$ after off-resonant excitation of lossless (**a**, $\gamma_{\text{cav}} = 0$ ps^{-1}) and lossy cavities (**b**, $\gamma_{\text{cav}} = 66.7$ ps^{-1}) filled with Tetracene molecules. The black lines represent $\langle z\rangle$ while the shaded area around the lines represents the root mean squared deviation (RMSD, *i.e.*, $\sqrt{\langle(z(t)-\langle z(t)\rangle)^2\rangle}$). Bottom panels: Mean Squared Displacement (MSD, *i.e.*, $\langle(z(t)-\langle z(0)\rangle)^2\rangle$) in the ideal lossless cavity (**c**) and the lossy cavity (**d**).

4.9.2 Methylene Blue

Because the broad absorption line-width of Tetracene reduces the duration of the ballistic propagation regime in Tetracene to a few fs, we also performed a simulation of Methylene Blue, which has an absorption line-width that is narrower than that of both Tetracene and Rhodamine. In Supplementary Figure 29, we show the wavepacket dynamics after exciting a wavepacket of LP states. Indeed, with a reduced overlap between the LP states and the molecular dark states, ballistic propagation is observed. As in Rhodamine (Supplementary Figure 2 in main text), the ballistic regime ends when the excitonic contributions to the total wave function become dominant, which happens around 75 fs for the cavity filled with Methylene Blue (Supplementary Figure 29**d**). In line with results for Rhodamine, the Mean Square Displacement then changes from a quadratic to linear time dependence, as indicated by the quadratic (magenta) and linear (cyan) fits in Supplementary Figure 30**b**. Thus, after on-resonant excitation of the cavity filled with Methylene Blue, propagation proceeds as in cavities containing Rhodamine or Tetracene, with a short ballistic phase when bright states are predominantly populated, followed by a diffusion phase when the population mostly resides inside the dark state manifold.

The additional simulations thus support the generality of a polariton transport mechanism based on reversible exchange of population between propagating polaritonic states and stationary dark states. Our results furthermore suggest that QM/MM simulations open up the possibility to systematically investigate the role of various molecular and cavity parameters on exciton transport. While further simulations are needed to disentangle these effects and formulate design rules for enhancing exciton transfer with optical resonators, the results we have obtained so far already suggest that this process can be controlled by tuning the overlap between the absorption line-width of the molecules and Rabi splitting of the strongly coupled light-matter systems.

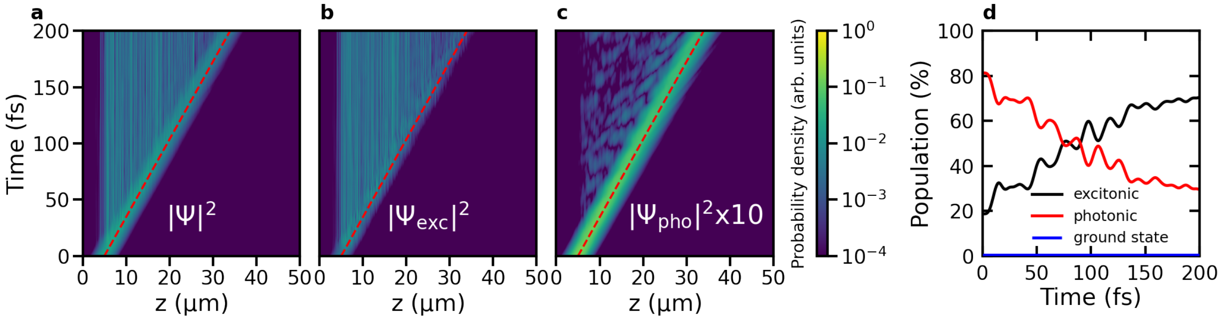

Supplementary Figure 29: Polariton propagation in cavities filled with Methylene Blue molecules after on-resonant excitation of a wavepacket of lower polariton (LP) states centered at $z = 5$ μm. Panels **a**, **b** and **c**: total probability density $|\Psi(z,t)|^2$, probability density of the molecular excitons $|\Psi_{\text{exc}}(z,t)|^2$ and of the cavity mode excitations $|\Psi_{\text{pho}}(z,t)|^2$, respectively, as a function of distance (z, horizontal axis) and time (vertical axis), in a cavity with perfect mirrors (*i.e.*, $\gamma_{\text{cav}} = 0$ ps^{-1}). The red dashed line indicates propagation at the maximum group velocity of the LP (145 μmps^{-1}). Panel **d**: Contributions of molecular excitons (black) and cavity mode excitations (red) to $|\Psi(z,t)|^2$ as a function of time in the perfect cavity. With no photon losses, no ground state population (blue) can build up.

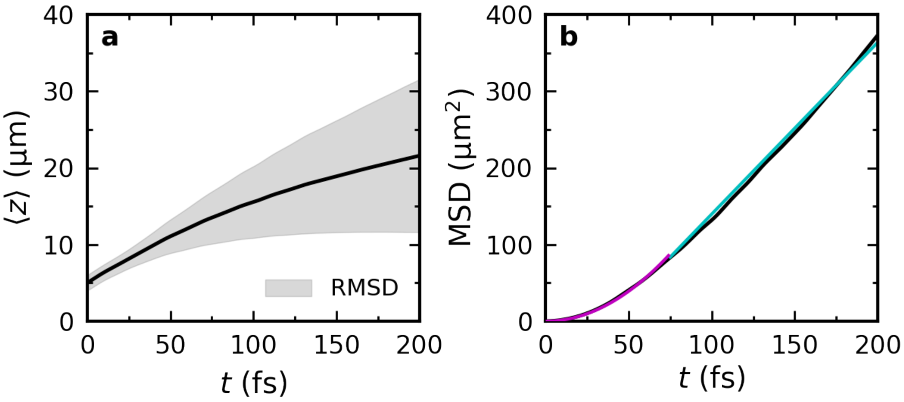

Supplementary Figure 30: Panel **a**: Expectation value of the position of the total time-dependent wavefunction $\langle z\rangle$=$\langle\Psi(z,t)|\hat{z}|\Psi(z,t)\rangle/\langle\Psi(z,t)|\Psi(z,t)\rangle$ after on-resonant excitation in a lossless cavity (*i.e.*, $\gamma_{\text{cav}} = 0$ ps^{-1}) filled with Methylene Blue molecules. The black line represents $\langle z\rangle$ while the shaded area around the line represents the root mean squared deviation (RMSD, *i.e.*, $\sqrt{\langle(z(t) - \langle z(t)\rangle)^2\rangle}$). Panel **b**: Mean squared displacement (MSD, *i.e.*, $\langle(z(t) - \langle z(0)\rangle)^2\rangle$) in the ideal lossless cavity. The magenta line is a quadratic fit to the Mean Squared Displacement and the cyan line is a linear fit.

5 Animations

Animations of the wavepackets in MPEG-4 format are available for download.

6 Supplementary References

(1) Forn-Díaz, P.; Lamata, L.; Rico, E.; Kono, J.; Solano, E. Ultrastrong coupling regimes of light-matter interaction. *Rev. Mod. Phys.* **2019**, *91*, 025005.

(2) Jaynes, E. T.; Cummings, F. W. Comparison of quantum and semiclassical radiation theories with application to the beam maser. *Proc. IEEE* **1963**, *51*, 89–109.

(3) Tavis, M.; Cummings, F. W. Approximate solutions for an N-molecule radiation-field Hamiltonian. *Phys. Rev.* **1969**, *188*, 692–695.

(4) Michetti, P.; Rocca, G. C. L. Polariton states in disordered organic microcavities. *Phys. Rev. B.* **2005**, *71*, 115320.

(5) Tichauer, R.; Feist, J.; Groenhof, G. Multi-scale Dynamics Simulations of Molecular Polaritons: the Effect of Multiple Cavity Modes on Polariton Relaxation. *J. Chem. Phys.* **2021**, *154*, 104112.

(6) Warshel, A.; Levitt, M. Theoretical studies of enzymatic reactions: Dielectric, electrostatic and steric stabilization of carbonium ion in the reaction of lysozyme. *J. Mol. Biol.* **1976**, *103*, 227–249.

(7) Boggio-Pasqua, M.; Burmeister, C. F.; Robb, M. A.; Groenhof, G. Photochemical reactions in biological systems: probing the effect of the environment by means of hybrid quantum chemistry/molecular mechanics simulations. *Phys. Chem. Chem. Phys.* **2012**, *14*, 7912–7928.

(8) Agranovich, V.; Gartstein, Y. Nature and Dynamics of Low-Energy Exciton Polaritons in Semiconductor Microcavities. *Phys. Rev. B* **2007**, *75*, 075302.

(9) Granucci, G.; Persico, M.; Toniolo, A. Direct semiclassical simulation of photochemical processes with semiempirical wave functions. *J. Chem. Phys.* **2001**, *114*, 10608–10615.

(10) Yarkony, D. R. Nonadiabatic Quantum Chemistry—Past, Present, and Future. *Chem. Rev.* **2012**, *112*, 481–498.

(11) Worth, G. A.; Cederbaum, L. A. Beyond Born-Oppenheimer: Molecular Dynamics Through a Conical Intersection. *Annu. Rev. Phys. Chem.* **2004**, *55*, 127–158.

(12) Azumi, T.; Matsuzaki, K. What does the term "vibronic coupling" mean? *Photochem. Photobiol.* **1977**, *25*, 315–326.

(13) Crespo-Otero, R.; Barbatti, M. Recent Advances and Perspectives on Nonadiabatic Mixed Quantum-Classical Dynamics. *Chem. Rev.* **2018**, *118*, 7026–7068.

(14) Luk, H.-L.; Feist, J.; Toppari, J. J.; Groenhof, G. Multiscale Molecular Dynamics Simulations of Polaritonic Chemistry. *J. Chem. Theory Comput.* **2017**, *13*, 4324–4335.

(15) Duan, Y.; Wu, C.; Chowdhury, S.; Lee, M. C.; Xiong, G. M.; Zhang, W.; Yang, R.; Cieplak, P.; Luo, R.; Lee, T.; Caldwell, J.; Wang, J. M.; Kollman, P. A point-charge force field for molecular mechanics simulations of proteins based on condensed-phase quantum mechanical calculations. *J. Comput. Chem.* **2003**, *24*, 1999–2012.

(16) Jorgensen, W. L.; Chandrasekhar, J.; Madura, J. D.; Impey, R. W.; Klein, M. L. Comparison of simple potential functions for simulatin liquid water. *J. Chem. Phys.* **1983**, *79*, 926–935.

(17) Berendsen, H.; Postma, J.; van Gunsteren, W.; la, A. D.; Haak, J. Molecular dynamics with coupling to an external bath. *J. Chem. Phys.* **1984**, *81*, 3684–3690.

(18) Hess, B.; Bekker, H.; Berendsen, H. J. C.; Fraaije, J. G. E. M. LINCS: A linear constraint solver for molecular simulations. *J. Comput. Chem.* **1997**, *18*, 1463–1472.

(19) Miyamoto, S.; Kollman, P. A. SETTLE: An analytical version of the SHAKE and RATTLE algorithms for rigid water molecules. *J. Comp. Chem.* **1992**, *18*, 1463–1472.

(20) Essmann, U.; Perera, L.; Berkowitz, M. L.; Darden, T.; Lee, H.; Pedersen, L. G. A smooth particle mesh Ewald potential. *J. Chem. Phys* **1995**, *103*, 8577–8592.

(21) Groenhof, G.; Climent, C.; Feist, J.; Morozov, D.; Toppari, J. J. Tracking Polariton Relaxation with Multiscale Molecular Dynamics Simulations. *J. Chem. Phys. Lett.* **2019**, *10*, 5476–5483.

(22) Hess, B.; Kutzner, C.; van der Spoel, D.; Lindahl, E. GROMACS 4: Algorithms for Highly Efficient, Load-Balanced, and Scalable Molecular Simulation. *J. Chem. Theory Comput.* **2008**, *4*, 435–447.

(23) Ufimtsev, I.; Martínez, T. J. Quantum Chemistry on Graphical Processing Units. 3. Analytical Energy Gradients and First Principles Molecular Dynamics. *J. Chem. Theory Comput.* **2009**, *5*, 2619–2628.

(24) Titov, A.; Ufimtsev, I.; Luehr, N.; Martínez, T. J. Generating Efficient Quantum Chemistry Codes for Novel Architectures. *J. Chem. Theory Comput.* **2013**, *9*, 213–221.

(25) Runge, E.; Gross, E. K. U. Density-Functional Theory for Time-Dependent Systems. *Phys. Rev. Lett* **1984**, *52*, 997–1000.

(26) Hirata, S.; Head-Gordon, M. Time-dependent density functional theory within the Tamm–Dancoff approximation. *Chem. Phys. Lett.* **1999**, *314*, 291–299.

(27) Becke, A. D. A new mixing of Hartree-Fock and local density-functional theories. *J. Chem. Phys.* **1993**, *98*, 1372.

(28) Yanai, T.; Tew, D. P.; Handy, N. C. A new hybrid exchange-correlation functional using the Coulomb-attenuating method (CAM-B3LYP. *Chem. Phys. Lett.* **2004**, *393*, 51–57.

(29) Dunning, T. H. Basis Functions for Use in Molecular Calculations. I. Contractions of (9s5p) Atomic Basis Sets for the First-Row Atoms. *J. Chem. Phys.* **1970**, *53*, 2823–2833.

(30) Roos, B. O. . Theoretical studies of electronically excited states of molecular systems using multiconfigurational perturbation theory. *Acc. Chem. Res.* **1999**, *32*, 137–144.

(31) Dunning, T. H. Gaussian basis sets for use in correlated molecular calculations. I. The atoms boron through neon and hydrogen. *J. Chem. Phys.* **1989**, *90*, 1007–1023.

(32) Granovsky, A. A. Extended multi-configuration quasi-degenerate perturbation theory: The new approach to multi-state multi-reference perturbation theory. *J. Chem. Phys.* **2011**, *134*, 214113.

(33) Frisch, M. J.; Trucks, G. W.; Schlegel, H. B.; Scuseria, G. E.; Robb, M. A.; Cheeseman, J. R.; Scalmani, G.; Barone, V.; Mennucci, B.; Petersson, G. A.; Nakatsuji, H.; Caricato, M.; Li, X.; Hratchian, H. P.; Izmaylov, A. F.; Bloino, J.; Zheng, G.; Sonnenberg, J. L.; Hada, M.; Ehara, M.; Toyota, K.; Fukuda, R.; Hasegawa, J.; Ishida, M.; Nakajima, T.; Honda, Y.; Kitao, O.; Nakai, H.; Vreven, T.; Montgomery, J. A., Jr.; Peralta, J. E.; Ogliaro, F.; Bearpark, M.; J.Heyd, J.; Brothers, E.; Kudin, K. N.; Staroverov, V. N.; Kobayashi, R.; Normand, J.; Raghavachari, K.; Rendell, A.; Burant, J. C.; Iyengar, S. S.; Tomasi, J.; Cossi, M.; Rega, N.; Millam, J. M.; Klene, M.; Knox, J. E.; Cross, J. B.; Bakken, V.; Adamo, C.; Jaramillo, J.; Gomperts, R.; Stratmann, R. E.; Yazyev, O.; Austin, A. J.; Cammi, R.; Pomelli, C.; Ochterski, J. W.; Martin, R. L.; Morokuma, K.; Zakrzewski, V. G.; Voth, G. A.; Salvador, P.; Dannenberg, J. J.; Dapprich, S.; Daniels, A. D.; Farkas, Ö.; Foresman, J. B.; Ortiz, J. V.; Cioslowski, J.; Fox, D. J. Gaussian 09 Revision D.1. Gaussian Inc. Wallingford CT 2009.

(34) Granovsky, A. A. Firefly version 8.2. Available at http://classic.chem.msu.su/gran/firefly/index.html.

(35) Baieva, S.; Hakamaa, O.; Groenhof, G.; Heikkilä, T. T.; Toppari, J. J. Dynamics of strongly coupled modes between surface plasmon polaritons and photoactive molecules: the effect of the Stokes shift. *ACS Photonics* **2017**, *4*, 28–37.

(36) Huang, W.; Lin, Z.; van Gunsteren, W. F. Validation of the GROMOS 54A7 Force Field with Respect to β-Peptide Folding. *Journal of Chemical Theory and Computation* **2011**, *7*, 1237–1243, PMID: 26610119.

(37) Berghuis, M. A.; Tichauer, R. H.; de Jong, L.; Sokolovskii, I.; Bai, P.; Ramezani, M.; Murai, S.; Groenhof, G.; Rivas, J. G. Controlling Exciton Propagation in Organic Crystals through Strong Coupling to Plasmonic Nanoparticle Arrays. *ACS Photonics* **2022**, *9*, 123.

(38) Bussi, G.; Donadio, D.; Parrinello, M. Canonical sampling through velocity rescaling. *J. Chem. Phys.* **2007**, *126*, 014101.

(39) Becke, A. D. Density-functional thermochemistry. V. Systematic optimization of exchange-correlation functionals. *J. Chem. Phys.* **1997**, *107*, 8554–8560.

(40) Schwartz, T.; Hutchison, J. A.; Leonard, J.; Genet, C.; Haacke, S.; Ebbesen, T. W. Polariton Dynamics under Strong Light-Molecule Coupling. *ChemPhysChem* **2013**, *14*, 125–131.

(41) George, J.; Wang, S.; Chervy, T.; Canaguier-Durand, A.; Schaeffer, G.; Lehn, J.-M.; Hutchison, J. A.; Genet, C.; Ebbesen, T. W. Ultra-strong coupling of molecular materials: spectroscopy and dynamics. *Faraday Discuss.* **2015**, *178*, 281–294.

(42) Rozenman, G. G.; Akulov, K.; Golombek, A.; Schwartz, T. Long-Range Transport of Organic Exciton-Polaritons Revealed by Ultrafast Microscopy. *ACS Photonics* **2018**, *5*, 105–110.

(43) Houdré, R.; Stanley, R. P.; Ilegems, M. Vacuum-field Rabi splitting in the presence of inhomogeneous broadening: Resolution of a homogeneous linewidth in an inhomogeneously broadened system. *Phys. Rev. A* **1996**, *53*, 2711–2715.

(44) Mony, J.; Climent, C.; Anne Ugleholdt Petersen, J. F., Kasper Moth-Poulsen; Börjesson, K. Photoisomerization Efficiency of a Solar Thermal Fuel in the Strong Coupling Regime. *Adv. Funct. Mater.* **2021**, *31*, 2010737.

(45) Frisch, M. J.; Trucks, G. W.; Schlegel, H. B.; Scuseria, G. E.; Robb, M. A.; Cheeseman, J. R.; Scalmani, G.; Barone, V.; Petersson, G. A.; Nakatsuji, H.; Li, X.; Caricato, M.; Marenich, A. V.; Bloino, J.; Janesko, B. G.; Gomperts, R.; Mennucci, B.; Hratchian, H. P.; Ortiz, J. V.; Izmaylov, A. F.; Sonnenberg, J. L.; Williams-Young, D.; Ding, F.; Lipparini, F.; Egidi, F.;

Goings, J.; Peng, B.; Petrone, A.; Henderson, T.; Ranasinghe, D.; Zakrzewski, V. G.; Gao, J.; Rega, N.; Zheng, G.; Liang, W.; Hada, M.; Ehara, M.; Toyota, K.; Fukuda, R.; Hasegawa, J.; Ishida, M.; Nakajima, T.; Honda, Y.; Kitao, O.; Nakai, H.; Vreven, T.; Throssell, K.; Montgomery, J. A., Jr.; Peralta, J. E.; Ogliaro, F.; Bearpark, M. J.; Heyd, J. J.; Brothers, E. N.; Kudin, K. N.; Staroverov, V. N.; Keith, T. A.; Kobayashi, R.; Normand, J.; Raghavachari, K.; Rendell, A. P.; Burant, J. C.; Iyengar, S. S.; Tomasi, J.; Cossi, M.; Millam, J. M.; Klene, M.; Adamo, C.; Cammi, R.; Ochterski, J. W.; Martin, R. L.; Morokuma, K.; Farkas, O.; Foresman, J. B.; Fox, D. J. Gaussian~16 Revision C.01. 2016; Gaussian Inc. Wallingford CT.

(46) Lidzey, D.; Bradley, D.; Armitage, A.; Walker, S.; Skolnick, M. Photon-Mediated Hybridization of Frenkel Excitons in Organic Semiconductor Microcavities. *Science* **2000**, *288*, 1620–1623.

(47) Tichauer, R. H.; Morozov, D.; Sokolovskii, I.; Toppari, J. J.; Groenhof, G. Identifying Vibrations that Control Non-Adiabatic Relaxation of Polaritons in Strongly Coupled Molecule-Cavity Systems. *J. Phys. Chem. Lett.* **2022**, *13*, 6259–6267.

(48) Pandya, R.; Chen, R. Y. S.; Gu, Q.; Sung, J.; Schnedermann, C.; Ojambati, O. S.; Chikkaraddy, R.; Gorman, J.; Jacucci, G.; Onelli, O. D.; Willhammar, T.; Johnstone, D. N.; Collins, S. M.; Midgley, P. A.; Auras, F.; Baikie, T.; Jayaprakash, R.; Mathevet, F.; Soucek, R.; Du, M.; Alvertis, A. M.; Ashoka, A.; Vignolini, S.; Lidzey, D. G.; Baumberg, J. J.; Friend, R. H.; Barisien, T.; Legrand, L.; Chin, A. W.; Yuen-Zhou, J.; Saikin, S. K.; Kukura, P.; Musser, A. J.; Rao, A. Microcavity-like exciton-polaritons can be the primary photoexcitation in bare organic semiconductors. *Nat. Commun.* **2021**, *12*, 6519.

(49) Freixanet, T.; Sermage, B.; Tiberj, A.; Planel, R. In-plane Propagation of Excitonic Cavity Polaritons. *Phys. Rev. B* **2000**, *61*, 7233.

(50) Flick, J.; Ruggenthaler, M.; Appel, H.; Rubio, A. Atoms and Molecules in Cavities: From

Weak to Strong Coupling in QED Chemistry. *Proc. Natl. Acad. Sci. USA* **2017**, *114*, 3026–3034.
