# Supplementary material for: Multi-scale molecular dynamics simulations of enhanced energy transfer in organic molecules under strong coupling
Source: Nat Commun. 2023 Oct 19;14:6613. doi: 10.1038/s41467-023-42067-y (PMC10587084; doi:10.1038/s41467-023-42067-y)
Supplement: Supplementary file 3 — Description of Additional Supplementary Files [file 41467_2023_42067_MOESM3_ESM.pdf]

Description of Additional Supplementary Files

File Name: Supplementary_Movie_1.mp4
Description: Total wavepacket after on-resonant excitation of a Gaussian wavepacket of LP states in a lossless cavity with 256 Rhodamines

File Name: Supplementary_Movie_2.mp4
Description: Excitonic contribution to wavepacket after on-resonant excitation of a Gaussian wavepacket of LP states in a lossless cavity with 256 Rhodamines

File Name: Supplementary_Movie_3.mp4
Description: Photonic contribution to wavepacket after on-resonant excitation of a Gaussian wavepacket of LP states in a lossless cavity with 256 Rhodamines

File Name: Supplementary_Movie_4.mp4
Description: Total wavepacket after on-resonant excitation of a Gaussian wavepacket of LP states in a lossless cavity with 512 Rhodamines

File Name: Supplementary_Movie_5.mp4
Description: Excitonic contribution to wavepacket after on-resonant excitation of a Gaussian wavepacket of LP states in a lossless cavity with 512 Rhodamines

File Name: Supplementary_Movie_6.mp4
Description: Photonic contribution to wavepacket after on-resonant excitation of a Gaussian wavepacket of LP states in a lossless cavity with 512 Rhodamines

File Name: Supplementary_Movie_7.mp4
Description: Total wavepacket after on-resonant excitation of a Gaussian wavepacket of LP states in a lossless cavity with 1024 Rhodamines

File Name: Supplementary_Movie_8.mp4
Description: Excitonic contribution to wavepacket after on-resonant excitation of a Gaussian wavepacket of LP states in a lossless cavity with 1024 Rhodamines

File Name: Supplementary_Movie_9.mp4
Description: Photonic contribution to wavepacket after on-resonant excitation of a Gaussian wavepacket of LP states in a lossless cavity with 1024 Rhodamines

File Name: Supplementary_Movie_10.mp4
Description: Total wavepacket after on-resonant excitation of a Gaussian wavepacket of LP states in a lossless cavity with 1024 Tetracene molecules

File Name: Supplementary_Movie_11.mp4
Description: Excitonic contribution to wavepacket after on-resonant excitation of a Gaussian wavepacket of LP states in a lossless cavity with 1024 Tetracene molecules

File Name: Supplementary_Movie_12.mp4
Description: Photonic contribution to wavepacket after on-resonant excitation of a Gaussian wavepacket of LP states in a lossless cavity with 1024 Tetracene molecules

File Name: Supplementary_Movie_13.mp4
Description: Total wavepacket after on-resonant excitation of a Gaussian wavepacket of LP states in a lossy cavity with 256 Rhodamines

File Name: Supplementary_Movie_14.mp4
Description: Excitonic contribution to wavepacket after on-resonant excitation of a Gaussian wavepacket of LP states in a lossy cavity with 256 Rhodamines

File Name: Supplementary_Movie_15.mp4
Description: Photonic contribution to wavepacket after on-resonant excitation of a Gaussian wavepacket of LP states in a lossy cavity with 256 Rhodamines

File Name: Supplementary_Movie_16.mp4
Description: Total wavepacket after on-resonant excitation of a Gaussian wavepacket of LP states in a lossy cavity with 512 Rhodamines

File Name: Supplementary_Movie_17.mp4
Description: Excitonic contribution to wavepacket after on-resonant excitation of a Gaussian wavepacket of LP states in a lossy cavity with 512 Rhodamines

File Name: Supplementary_Movie_18.mp4
Description: Photonic contribution to wavepacket after on-resonant excitation of a Gaussian wavepacket of LP states in a lossy cavity with 512 Rhodamines

File Name: Supplementary_Movie_19.mp4
Description: Total wavepacket after on-resonant excitation of a Gaussian wavepacket of LP states in a lossy cavity with 1024 Rhodamines

File Name: Supplementary_Movie_20.mp4
Description: Excitonic contribution to wavepacket after on-resonant excitation of a Gaussian wavepacket of LP states in a lossy cavity with 1024 Rhodamines

File Name: Supplementary_Movie_21.mp4
Description: Photonic contribution to wavepacket after on-resonant excitation of a Gaussian wavepacket of LP states in a lossy cavity with 1024 Rhodamines

File Name: Supplementary_Movie_22.mp4
Description: Total wavepacket after on-resonant excitation of a Gaussian wavepacket of LP states in a lossy cavity with 1024 Tetracene molecules

File Name: Supplementary_Movie_23.mp4
Description: Excitonic contribution to wavepacket after on-resonant excitation of a Gaussian wavepacket of LP states in a lossy cavity with 1024 Tetracene molecules

File Name: Supplementary_Movie_24.mp4
Description: Photonic contribution to wavepacket after on-resonant excitation of a Gaussian wavepacket of LP states in a lossy cavity with 1024 Tetracene molecules

File Name: Supplementary_Movie_25.mp4
Description: Total wavepacket after off-resonant excitation of a single molecule in a lossless cavity with 256 Rhodamines

File Name: Supplementary_Movie_26.mp4
Description: Excitonic contribution to wavepacket after off-resonant excitation of a single molecule in a lossless cavity with 256 Rhodamines

File Name: Supplementary_Movie_27.mp4
Description: Photonic contribution to wavepacket after off-resonant excitation of a single molecule in a lossless cavity with 256 Rhodamines

File Name: Supplementary_Movie_28.mp4
Description: Total wavepacket after off-resonant excitation of a single molecule in a lossless cavity with 512 Rhodamines

File Name: Supplementary_Movie_29.mp4
Description: Excitonic contribution to wavepacket after off-resonant excitation of a single molecule in a lossless cavity with 512 Rhodamines

File Name: Supplementary_Movie_30.mp4
Description: Photonic contribution to wavepacket after off-resonant excitation of a single molecule in a lossless cavity with 512 Rhodamines

File Name: Supplementary_Movie_31.mp4
Description: Total wavepacket after off-resonant excitation of a single molecule in a lossless cavity with 1024 Rhodamines

File Name: Supplementary_Movie_32.mp4
Description: Excitonic contribution to wavepacket after off-resonant excitation of a single molecule in a lossless cavity with 1024 Rhodamines

File Name: Supplementary_Movie_33.mp4
Description: Photonic contribution to wavepacket after off-resonant excitation of a single molecule in a lossless cavity with 1024 Rhodamines

File Name: Supplementary_Movie_34.mp4
Description: Total wavepacket after off-resonant excitation of a single molecule in a lossless cavity with 1024 Tetracene molecules

File Name: Supplementary_Movie_35.mp4
Description: Excitonic contribution to wavepacket after off-resonant excitation of a single molecule in a lossless cavity with 1024 Tetracene molecules

File Name: Supplementary_Movie_36.mp4
Description: Photonic contribution to wavepacket after off-resonant excitation of a single molecule in a lossless cavity with 1024 Tetracene molecules

File Name: Supplementary_Movie_37.mp4
Description: Total wavepacket after off-resonant excitation of a single molecule in a lossy cavity with 256 Rhodamines

File Name: Supplementary_Movie_38.mp4
Description: Excitonic contribution to wavepacket after off-resonant excitation of a single molecule in a lossy cavity with 256 Rhodamines

File Name: Supplementary_Movie_39.mp4
Description: Photonic contribution to wavepacket after off-resonant excitation of a single molecule in a lossy cavity with 256 Rhodamines

File Name: Supplementary_Movie_40.mp4
Description: Total wavepacket after off-resonant excitation of a single molecule in a lossy cavity with 512 Rhodamines

File Name: Supplementary_Movie_41.mp4
Description: Excitonic contribution to wavepacket after off-resonant excitation of a single molecule in a lossy cavity with 512 Rhodamines

File Name: Supplementary_Movie_42.mp4
Description: Photonic contribution to wavepacket after off-resonant excitation of a single molecule in a lossy cavity with 512 Rhodamines

File Name: Supplementary_Movie_43.mp4
Description: Total wavepacket after off-resonant excitation of a single molecule in a lossy cavity with 1024 Rhodamines

File Name: Supplementary_Movie_44.mp4
Description: Excitonic contribution to wavepacket after off-resonant excitation of a single molecule in a lossy cavity with 1024 Rhodamines

File Name: Supplementary_Movie_45.mp4
Description: Photonic contribution to wavepacket after off-resonant excitation of a single molecule in a lossy cavity with 1024 Rhodamines

File Name: Supplementary_Movie_46.mp4
Description: Total wavepacket after off-resonant excitation of a single molecule in a lossy cavity with 1024 Tetracene molecules

File Name: Supplementary_Movie_47.mp4
Description: Excitonic contribution to wavepacket after off-resonant excitation of a single molecule in a lossy cavity with 1024 Tetracene molecules

File Name: Supplementary_Movie_48.mp4
Description: Photonic contribution to wavepacket after off-resonant excitation of a single molecule in a lossy cavity with 1024 Tetracene molecules
